# Metabolomic Profile of *Vaccinium corymbosum* Leaves: Exploiting Diversity Among Ten Different Cultivars

**DOI:** 10.3390/foods14162846

**Published:** 2025-08-17

**Authors:** Tânia Ribeiro, Manuela E. Pintado, Clara Sousa

**Affiliations:** CBQF—Centro de Biotecnologia e Química Fina—Laboratório Associado, Escola Superior de Biotecnologia, Universidade Católica Portuguesa, Rua Diogo Botelho 1327, 4169-005 Porto, Portugal; tribeiro@ucp.pt (T.R.); mpintado@ucp.pt (M.E.P.)

**Keywords:** blueberries, mass spectrometry, liquid chromatography, secondary metabolites, plant leaves

## Abstract

Blueberry (*Vaccinium corymbosum*) leaves are often discarded as agricultural by-products despite their potential abundance in bioactive compounds. However, comprehensive knowledge of their phytochemical profile remains limited, especially at the cultivar level. To address this gap, this study performed untargeted metabolomic profiling of blueberry leaves from ten cultivars using UHPLC-QTOF MS. Metabolites were annotated using high-resolution mass spectrometry and MS/MS fragmentation patterns. Multivariate statistical techniques were employed to investigate inter-cultivar variability and identify distinctive metabolites. A total of 76 metabolites were discovered, with 64 being confidently annotated and grouped into ten main phytochemical classes. The relative abundances of phenolic acids, flavonols, and flavan-3-ols varied significantly among cultivars. Several metabolites were annotated for the first time in *V. corymbosum* leaves, such as miscanthoside, glucoliquiritin, apiin, khelloside, and aromadendrin. These metabolites are known in other plants for their biological activities, demonstrating blueberry leaves’ bioactive potential. This study highlights the importance of untargeted metabolomic approaches in elucidating the biochemical diversity of plant matrices. The metabolomic data revealed significant cultivar-specific variations and novel bioactive metabolite annotation. These findings establish a complete phytochemical fingerprint for each cultivar, providing a basis for future research to validate key metabolites’ biological activities and support the valorisation of *V. corymbosum* leaves.

## 1. Introduction

*Vaccinium corymbosum* of the Ericaceae family is a North American species of blueberry holding significant relevance across the economic, health, and industrial sectors. This species is widely known for its rich nutritional profile and health benefits. Blueberries, like many other berries, are rich in antioxidants, particularly polyphenols, which have been linked to improved cognitive function, cardiovascular health, and reduced risk of chronic diseases such as type 2 diabetes and certain cancers [1,2]. The cosmetic industry also utilises blueberry extracts in skincare formulations due to their antioxidant properties [3]. Their antibacterial activity against specific species such as Staphylococcus aureus, Listeria monocytogenes, and Bacillus cereus has also been reported [4]. *V. corymbosum* boasts a remarkable diversity of cultivars, each exhibiting quite unique characteristics. These variations span crucial traits such as ripening time (early, mid, or late season), fruit size, flavour profile (sweet, tart, or aromatic), yield potential, growth habit (upright, spreading, or compact), winter hardiness, and disease resistance. This variability allows growers to choose cultivars best suited to their specific climate, soil conditions, market demands, and desired harvest window. It is easily inferred that all these differences between cultivars are mirrored in the plant metabolome, including their leaves [5]. Indeed, interest in the leaves of *V. corymbosum* shrubs, due to their proven potential as a source of bioactive compounds [6], has increased, and some works have been published aiming at its characterisation [7,8,9]. Routray and Orsat [7] analysed two *V. corymbosum* cultivars, Elliot and Nelson, and concluded that the latter possesses the highest content of monomeric anthocyanins, using a combination of ethanol and citric acid as the extraction solvent. Akšić et al. [8] characterised three cultivars (‘Duke’, Nui, and Bluecrop) and concluded that 5-*O*-caffeoylquinic acid was the most abundant phenolic in blueberry leaves when MeOH/H_2_O (70:30) plus 0.1% of HCl was used as the extraction solvent. Venskutonis et al. [9] compared seven cultivars and denoted that rutin, chlorogenic, and quinic acid concentrations for the same cultivar were highly dependent on the extraction solvent. The full characterisation of the metabolic profile of *V. corymbosum* cultivars’ leaves will capitalise on its use; however, most of the published works used few cultivars and diverse extraction solvents. To the best of our knowledge, the most complete study was undertaken by Wu and colleagues [10]. These authors claimed the characterisation of 73 different cultivars; however, these cultivars were grouped, and the metabolomic profile was presented for each group instead of per cultivar individually, avoiding a deep knowledge of cultivar diversity.

In this study, the metabolomic profile of *V. corymbosum* leaves of 14 plants belonging to 10 different cultivars was obtained by mass spectrometry in an ultra-high-performance liquid chromatography–quadrupole time-of-flight mass spectrometer (UHPLC-QTOF MS). Metabolites were annotated with their exact masses and experimentally generated fragments with the aid of online-available and licensed software. The annotated metabolites were further quantified (semi-quantitative approach), and differences between cultivars were discussed. Additionally, a principal component analysis was performed, and the corresponding variables’ importance on projection (VIP) scores were obtained allowing the annotation of the most relevant metabolites in the leaves of each cultivar.

## 2. Materials and Methods

### 2.1. The Samples

*V. corymbosum* leaves of 14 distinct plants belonging to 10 cultivars [‘Legacy’ (*n* = 3); ‘Duke’ (*n* = 3); ‘Liberty’ (*n* = 1); ‘Aurora’ (*n* = 1); ‘Camellia’ (*n* = 1); ‘Spartan’ (*n* = 1); ‘Susyblue’ (*n* = 1); ‘Star’ (*n* = 1); ‘Rabbiteye’ (*n* = 1); ‘Centrablue’ (*n* = 1)] were collected in spring (April) in the north of Portugal, in Arouca (40.92481384287574° N,−8.252706527709961° E). All cultivars were grown under the same geographical, soil, climatic, and cultivation conditions to ensure environmental uniformity. Leaf samples were collected in the early morning and promptly transported to the laboratory in proper bags in a refrigerated container. Immediately after arriving at the laboratory, the samples were cleaned with water, dried with paper towels, and further lyophilised. Lyophilised samples were ground and stored at −80 °C until the UHPLC-QTOF MS experiments (for no more than two weeks).

### 2.2. Extraction Procedure

Lyophilised leaves were taken from the refrigerator and left for 30 min in the laboratory, allowing them to reach room temperature. The samples were ground, and an average of fifty milligrams of each sample was added (in triplicate) to a 50 mL vial after adding 5 mL of pure ethanol (MS grade). Vials were sonicated for 15 min and further centrifuged. The supernatants were filtered through a 0.22 mm syringe filter, transferred to vials, and further analysed. Extracts were obtained in triplicate.

### 2.3. UHPLC-QTOF MS

The MS/MS experiments were conducted using ultra-high-performance liquid chromatography–quadrupole time-of-flight mass spectrometry (UHPLC-QTOF MS) equipment. The chromatographic separations were undertaken in a UHPLC UltiMate 3000 Dionex system (Thermo Fisher Scientific Inc., Waltham, MA, USA), and the mass experiments in a mass spectrometer from Bruker Daltonics Impact II, Bremen, Germany, equipped with an electrospray ionisation (ESI) source. The mass spectrometer was operated in negative ion mode, and data were recorded under the following ESI source parameters: capillary voltage, 3.5 kV; end plate offset, 500 V; nebuliser, 0.4 Bar; dry gas, 4.0 L/min; dry temperature, 180 °C; Funnel 1 RF, 400 Vpp; Funnel 2 RF, 600 Vpp; hexapole, 700 Vpp; quadrupole ion energy, 5.0 eV; collision energy, 10.0 eV; transfer time, 120.0 µs. The QTOF mass analyser scanned masses in the range of 50–1000 Da.

Extracts (5 μL) were injected on an Acclaim RSLC 120 C18 column (100 mm × 2.1 mm, 2.2 µm) (Thermo Fisher Scientific Inc., Sunnyvale, CA, USA). The mobile phase was a binary solvent system consisting of (A) water acidified with formic acid (0.1%) and (B) acetonitrile acidified with formic acid (0.1%). The gradient (0–25 min with 5–90% of B in gradual steps) was eluted at a flow rate of 0.25 mL/min at 40 °C. Each sample was run in duplicate.

### 2.4. Data Analysis

The data were analysed with free online-available (Compound Crawler 2.0, MetFrag v2.4.5 and MetaboAnalyst 5.0) and/or licensed software (Smart Formula 3D, DataAnalysis 4.4 and Metaboscape 2023). Raw mass spectra were calibrated and processed in Bruker Metaboscape software. Only features with MS/MS spectra were considered. The feature table was further subjected to annotation by comparison with spectral libraries (Bruker NIST 2020 MSMS Spectral Library; MassBank_NIST.msp and MassBank2RIKEN.msp). The putative compounds were annotated with a tolerance of 5.0 ppm. After the putative annotations, in silico fragmentation of all the compounds were generated, and theoretical vs. experimental fragmentation patterns were compared. The final annotation was assumed for metabolites presenting a score ≥0.99 in MetFrag software. The proposed metabolite annotation was also checked and compared with the available literature.

## 3. Results

### 3.1. Metabolic Profiling of V. corymbosum Cultivars by HPLC-QTOF MS

The metabolome of *V. corymbosum* cultivars was characterised by the LC-MS data, including retention time, molecular formula, experimental exact mass and *m*/*z* fragments generated, mSigma values (which are the rate for the agreement of the theoretical and measured isotopic pattern of the mass peak of interest; lower numbers correspond to better fits and higher annotation confidence), and accuracy (ppm). A total of 76 metabolites were detected in the ethanolic leaf extract, 64 of which were annotated (Table 1 and Table S1), and 12 were non-annotated (Table S2).

**Figure 1:**
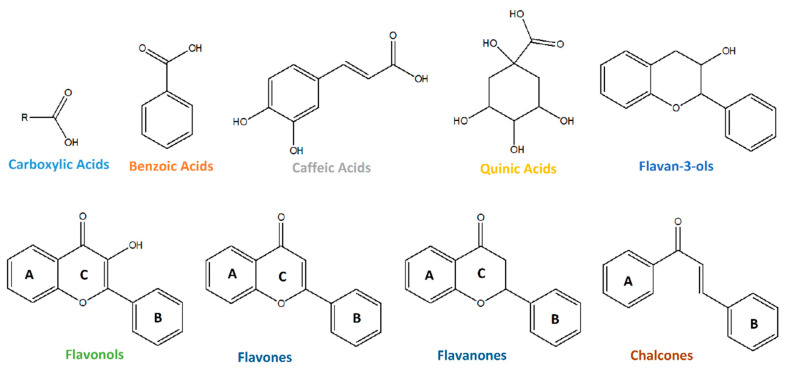
Compounds classes annotated in the ethanolic leaf extracts of *V. corymbosum* cultivars.

The annotated metabolites (64) belong to distinct compound classes: carboxylic acids (10/65); benzoic acids (14/65); caffeic acids (3/65); quinic acids (10/65); flavan-3-ols (5/65); flavonols (13/65); flavones and flavanones (4/65); chalcones (2/65); and 3/65 belonging to other classes (Figure 1).

**Carboxylic acids:** Compound **C1** was annotated as succinic acid, presenting the *m*/*z* 73 fragment, which corresponds to the typical fragmentation ion of carboxylic acids (the loss of the CO_2_ unit, [M−H−44]). The unique fragment (*m*/*z* 73) observed for succinic acid agrees with previous studies [11]. Compound **C2** was annotated as citramalic acid. The observed fragments *m*/*z* 87, *m*/*z* 85, and *m*/*z* 129 are due to the [C_3_H_5_O_3_−H]^−^H^−^, [C_4_H_4_O_2_+H]−H^−^, and [C_5_H_6_O_4_−H]−H^−^ moieties, respectively. Compound **C3** was annotated as hydroxypentanoic acid. Fragments of *m*/*z* 73 (typical loss of CO_2_) and *m*/*z* 59 ([C_2_H_3_O_2_−H]^−^ moiety) were observed. Compounds **C4** and **C5** were annotated as coumaric acids. Both present a fragment ion *m*/*z* 119 due to the loss of CO_2_ [M−H−44], and compound **C4** presents an additional fragment ion at *m*/*z* 71 from the [C_3_H_3_O_2_]^−^ moiety. Mekky and colleagues [12] have annotated m- and *p*-coumaric acids in sesame cake and, according to the standards used, proved that when using a C18 column with acidified water and acetonitrile as mobile phases, *p*-coumaric acid was first eluted. This suggests that compound **C4** is *p*-coumaric acid and compound **C5** is *m*-coumaric acid. Compound **C6** was annotated as azelaic acid, presenting m/125 and *m*/*z* 169, *m*/*z* 123, and *m*/*z* 143 (loss of CO_2_) fragments due to [C_8_H_12_O+H]^−^, [C_9_H_14_O_3_]−H^−^, [C_8_H_12_O]−H^−^, and [C_8_H_14_O_2_+H]^−^ moieties, respectively. Compound **C7** was annotated as undecanoic acid with *m*/*z* 197 and *m*/*z* 153 fragments due to the [C_11_H_18_O_3_+H]−H^−^ and [C_10_H_16_O+H]^−^ moieties, respectively. Compounds **C8** and **C9** were annotated as hydroxydodecanoic acids. Both present the *m*/*z* 169 fragment due to the [C_11_H_22_O]−H^−^ moiety. The remaining fragments of both isomers are compatible with the hydroxypentanoic structure: *m*/*z* 87 ([C_3_H_5_O_3_−H]−H^−^), *m*/*z* 171 ([C_9_H_17_O_3_−H]−H^−^), *m*/*z* 197 ([C_12_H_22_O_2_]−H^−^), and *m*/*z* 199 ([C_11_H_21_O_3_−H]−H^−^). Compound **C10** was annotated as a furancarboxylic acid, with fragments of *m*/*z* 209.155 ([C_13_H_20_O_2_+H]^−^), *m*/*z* 210 ([C_13_H_22_O_2_]^−^), *m*/*z* 59 ([C_2_H_2_O_2_+H]^−^), *m*/*z* 209.120 ([C1_2_H_15_O_3_+2H]^−^), and *m*/*z* 89 ([C_3_H_3_O_3_+2H]^−^).

**Benzoic acids:** Compounds **B11**, **B12**, **B13**, and **B14** present a main fragment of *m*/*z* 93 corresponding to the typical loss of CO_2_ [M−H−44]^−^. Compounds **B12**, **B13**, and **B14** present nearly the same fragmentation patterns, which is suggestive of *o*-, *m*-, and *p*-hydroxybenzoic acids. Compound **B11** presented a quite different fragmentation pattern, suggesting a di-hydroxybenzaldehyde, namely the protocatechuic aldehyde (all the fragments can be explained and correlated with this compound). Compounds **B15** and **B16** were annotated as di-hydroxybenzoic acids according to the fragmentation pattern: *m*/*z* 108 from the [C_6_H_4_O_2_]^−^, *m*/*z* 109 from the [C_6_H_4_O_2_+H]^−^, and *m*/*z* 123 from the [C_7_H_5_O_2_+2H]^−^ moieties. Compounds **B17**, **B18**, and **B19** were annotated as tri-hydroxybenzoic acids, with all the fragments being explained and compatible with such a structure. Compound **B17** was considered to be gallic acid (a tri-hydroxybenzoic acid) according to the exact mass and unique *m*/*z* 125 fragment. It should be noted that gallic acid was already annotated in previous studies and reported with *m*/*z* 125 as the unique fragment and an exact mass of [M−H]^−^ 162.0142 [11]. Compounds **B20**, **B21**, and **B22** were annotated as hydroxybenzoic acid hexosides. The typical fragments of *m*/*z* 137 related to the loss of the glucosyl moiety [M−H−162] was observed in the three compounds. Compounds **B20** and **B22** additionally possess the *m*/*z* 93 fragment typical of the [C_6_H_5_O]^−^ moiety, and compound **B21** possesses the fragments *m*/*z* 179, *m*/*z* 239, and *m*/*z* 151. The three isomers are compatible with a glucosyl moiety linked to both hydroxyl groups (that of the carboxylic acid or the free one in the benzene ring). Compound **B23** was annotated as a di-hydroxybenzoic acid hexoside, with the main fragments being *m*/*z* 152 and *m*/*z* 108 due to the loss of the glucosyl moiety and the benzene ring with both oxygen atoms from the hydroxyl groups. Compound **B24** was annotated as vanillic acid hexoside, with all fragments explained and compatible with the proposed structure, including the main fragment *m*/*z* 167, corresponding to the loss of the glucosyl moiety [M−H−162]^−^.

**Caffeic acids:** Compound **Caf25** possesses the *m*/*z* 135 main fragment due to the loss of carbon dioxide [C_8_H_6_O_2_+H]^−^ and *m*/*z* 134 ([C_8_H_6_O_2_]^−^), being annotated as caffeic acid (cis-/trans-3,4-dihydroxycinnamic acid) in accordance with the literature [8,13], Bruker Metaboscape software, and MassBank record MSBNK-RIKEN-PR100533. Compound **Caf26** was annotated as a caffeic acid isomer due to the exact mass and the presence of *m*/*z* 135 (similar to that encountered in compound **Caf25**) and *m*/*z* 137 from the [C_8_H_7_O_2_+2H]^−^ moiety. Compound **Caf27** was annotated as caffeic acid hexoside. The exact mass and fragments of *m*/*z* 179 (loss of the hexoside moiety), *m*/*z* 135 (loss of CO_2_ from the caffeic acid moiety), and *m*/*z* 181 (from the [C_9_H_9_O_4_]^−^ moiety) confirm the identification.

**Quinic acid derivatives:** Compounds **Q28**, **Q29**, **Q30**, and **Q31** were annotated as caffeoyl quinic acid isomers. Typical fragments of *m*/*z* 191 and *m*/*z* 179 due to quinic acid and caffeoyl moieties, respectively, and of *m*/*z* 173 (quinic acid moiety water loss) and *m*/*z* 135 (caffeoyl moiety carbon dioxide loss) were annotated. Compound **Q29** uniquely presents the *m*/*z* 191 fragment, which suggests 5-caffeoyl quinic acid. Indeed, Venskutonis et al. [9] proved that in a C18 column eluted with a gradient of water + formic acid followed by an organic solvent, 3-caffeoyl quinic acid is first eluted, then the isomer 5-caffeoyl quinic acid, which is in accordance with our observations. Compounds **Q32**, **Q33**, and **Q34** were annotated as coumaroyl quinic acids. Fragments of *m*/*z* 191 and *m*/*z* 173 are associated with the quinic acid moiety (as explained above), the fragment *m*/*z* 163 corresponds to the coumaroyl moiety, while the fragment *m*/*z* 119 reflects the loss of carbon dioxide of the coumaroyl moiety. Compound **Q35** was annotated as feruloylquinic acid due to its exact mass and typical fragments of *m*/*z* 191 and *m*/*z* 173 (quinic acid moiety and its loss of water), *m*/*z* 193 due to the feruloyl moiety, and *m*/*z* 93 (aromatic ring from the feruloyl moiety with the oxygen atom from the hydroxyl group). Compound **Q36** was annotated as dicaffeoylquinic acid. The exact mass and typical fragments of *m*/*z* 353 (monocaffeoylquinic acid moiety), *m*/*z* 191, *m*/*z* 173 (quinic acid moiety and its loss of water), and *m*/*z* 179 (caffeoyl unit) are compatible with a dicaffeoylquinic acid structure. Compound **Q37** was annotated as a feruloylcaffeoylquinic acid due to its exact mass and typical fragments of *m*/*z* 367 (loss of the caffeoyl unit) and *m*/*z* 193, *m*/*z* 191 *m*/*z* 353, *m*/*z* 173, and *m*/*z* 179 (due to the already-explained structures).

**Flavan-3-ols:** Five catechin derivatives were annotated in the extracts. Compounds **Fla38** and **Fla39** were annotated as gallocatechin/epigallocatechin and catechin/epicatechin, respectively, based on their exact masses and fragmentation patterns. Compound **Fla38** presents the typical gallocatechin/epigallocatechin fragmentation pattern with the main fragment of *m*/*z* 125 ([C_6_H_5_O_3_]^−^) and three additional ones (*m*/*z* 165, 167, and 137 of similar intensities from [C_8_H_6_O_4_]−H^−^, [C_8_H_8_O_4_]−H^−^, and [C_7_H_6_O_3_]−H^−^ moieties, respectively) reported previously [13] and compatible with MassBank records BS003906 (gallocatechin) and BS003952 (epigallocatechin). Regarding catechin/epicatechin, compound **Fla39**, fragments of *m*/*z* 245 and *m*/*z* 203 from [C_14_H_11_O_4_+2H]^−^ and [C_12_H_9_O_3_+2H]^−^, respectively, were observed herein and reported in other studies [13], as is typical of these compounds. Three trimeric catechin isomers (compounds **Fla40**, **Fla41**, and **Fla42**) were annotated through their exact masses and fragmentation patterns as type A/B (one of the units bonded to the central one with an A-type bonding, and the other bonded to the central one with a B-type bonding). Hokkanen et al. [13] reported the same fragmentation pattern obtained herein for compounds **Fla41** and **Fla42** in their studies: *m*/*z* 711 due to RDA fragmentation; *m*/*z* 451 loss of the extension A-type unit plus pholoroglucinol from the central unit; *m*/*z* 411 extension unit and pholoroglucinol from the central unit; *m*/*z* 289 the terminal unit; and *m*/*z* 573 loss of the B-type terminal unit. A different study [14] has also annotated two procyanin type A/B isomers through their exact masses and the *m*/*z* 298 fragment.

**Flavonols:** Compound **Flo43** was annotated as aromadendrin. The observed fragments of *m*/*z* 259, *m*/*z* 143, *m*/*z* 125, and *m*/*z* 152 from the [C_14_H_12_O_5_]−H^−^, [C_14_H_10_O_4_+H]^−^, [C_6_H_4_O_3_+H]^−^, and [C_7_H_4_O_4_]^−^ moieties, respectively, are compatible with the proposed compound. Compound **Flo44** was annotated as isorhamnetin. Fragments of *m*/*z* 300 ([C_15_H_9_O_7_]−H^−^) and *m*/*z* 301 ([C_15_H_9_O_7_]^−^), due to the loss of the CH_4_ and CH_3_· moieties, respectively, and *m*/*z* 151 from ring C cleavage between positions 1 and 3 ([C_7_H_4_O_4_]−H^−^) were observed. Compound **Flo45** was annotated as kaempferol-7-*O*-rhamnoside. The main fragments of *m*/*z* 285 ([C_15_H_9_O_6_]^−^) and *m*/*z* 284 ([C_15_H_9_O_6_]−H^−^), due to the loss of the rhamnose moiety and *m*/*z* 191 ([C_7_H_9_O_6_+2H]^−^), were observed. Compound **Flo46** was annotated as myricetin-3-hexoside. The typical loss of the hexosyl moiety (−162) was observed through the presence of the fragment *m*/*z* 317 ([C_15_H_9_O_8_]^−^). Additional fragments of *m*/*z* 191 and *m*/*z* 301 from the [C_7_H_11_O_6_]^−^ and [C_15_H_9_O_7_]^−^ moieties were also observed. Compound **Flo47** was annotated as syringetin-3-hexoside due to the presence of the typical fragment of *m*/*z* 344 ([C_17_H_13_O_8_]−H^−^) due to the loss of the hexosyl moiety (−162). Compound **Flo48** was annotated as quercetin-3-*O*-pentosylpentosilpentoside. The presence of the typical fragment of *m*/*z* 300 ([C_15_H_9_O_7_]−H^−^), due to the loss of the sugar moieties, and the additional fragments of *m*/*z* 463 and *m*/*z* 191 from [C_21_H_18_O_12_+H]^−^ and [C_7_H_12_O_6_]−H^−^ moieties, respectively, confirms the identification. Compounds **Flo49** and **Flo50** were annotated as possessing a flavonol base with a 3-*O*-hexoside-hexoside moiety. The loss of the sugar moieties was the main fragment observed (*m*/*z* 285 from [C_15_H_9_O_6_]^−^ and *m*/*z* 284 from [C_15_H_9_O_6_]−H^−^). Compound **Flo49** was tentatively annotated as kaempferol 3-*O*-rutinoside with a score of 1.0 in Bruker MetaboScape software. Additional *m*/*z* 463 and *m*/*z* 327 fragments from [C_15_H_19_O_12_]^−^ and [C_21_H_12_O_7_]−H^−^ moieties were also observed. Compound **Flo51** was annotated as rutin through the exact mass and the typical unique fragment of *m*/*z* 300 from the [C_15_H_9_O_7_]−H^−^ moiety. Compound **Flo52** was annotated as quercetin 3-gentiobioside, with the five most intense fragments perfectly explained, namely, *m*/*z* 445 ([C_21_H_19_O_11_−H]−H^−^), *m*/*z* 463 ([C_21_H_19_O_12_]H^−^), *m*/*z* 301 ([C_15_H_9_O_7_]^−^), *m*/*z* 464 ([C_21_H_19_O_12_+H]^−^), and *m*/*z* 300 ([C_15_H_9_O_7_]−H^−^). Compound **Flo53** was annotated as possessing a flavonol base plus three sugar moieties. A higher identification score (1.0) was obtained for helieianeoside B in Bruker MetaboScape software, with the five most intense fragments compatible with the proposed identification: *m*/*z* 300 ([C_15_H_9_O_7_]−H^−^), *m*/*z* 191 ([C_7_H_12_O_6_]−H^−^), *m*/*z* 353 ([C_16_H_15_O_9_+2H]^−^), *m*/*z* 417 ([C_21_H_19_O_9_+2H]^−^), and *m*/*z* 178 ([C_8_H_4_O_5_]−H^−^). Compound **Flo54** was annotated as kaempferol-3-*O*-rutinoside-7-*O*-β-D-glucopyranoside with a score of 1.0 in Bruker MetaboScape software (both fragments explained): *m*/*z* 593 ([C_27_H_29_O_15_]^−^) and *m*/*z* 285 ([C_15_H_9_O_6_]^−^). The fragments and exact mass were also in accordance with the literature [10]. Compound **Flo55** was annotated as variabiloside with a score of 1.0 in Bruker MetaboScape software (both fragments explained): *m*/*z* 593 ([C_30_H_25_O_13_]^−^) and *m*/*z* 285 ([C_15_H_9_O_6_]^−^).

**Flavones and flavanones:** Flavones and flavanones differ in the saturation degree of the carbon–carbon bond of ring C. Four compounds were annotated: three flavanones (**Flav56**, **Flav 57**, and **Flav59**) and one flavone (**Flav58**). Compound **Flav56** was annotated as 3,9-dihydroeucomin through the presence of its typical fragments at *m*/*z* 179 ([C_9_H_7_O_4_]^−^), *m*/*z* 151 ([C_7_H_4_O_4_]−H^−^), *m*/*z* 135 ([C_8_H_8_O_2_]−H^−^), *m*/*z* 165 ([C_8_H_6_O_4_]−H^−^), *m*/*z* 121 ([C_7_H_4_O_2_+H]^−^), *m*/*z* 229 ([C_13_H_11_O_4_−H]−H^−^), and *m*/*z* 149 ([C_9_H_9_O_2_]^−^). Compound **Flav57** was annotated as miscanthoside, with fragments at *m*/*z* 287 (C_15_H_11_O_6_]^−^), *m*/*z* 191 ([C_7_H_11_O_6_]^−^), *m*/*z* 151 ([C_7_H_3_O_4_]^−^), *m*/*z* 257 ([C_15_H_11_O_4_+2H]^−^), *m*/*z* 301 [C_15_H_10_O_7_−H]^−^, and *m*/*z* 135 ([C_8_H_8_O_2_]−H^−^). Compound **Flav58** was annotated as apiin though its fragments at *m*/*z* 191 ([C_7_H_12_O_16_]−H^−^) and at *m*/*z* 353 ([C_16_H_15_O_9_+2H]^−^). Compound **Flav59** was annotated as glucoquiritin through its fragments at *m*/*z* 417 ([C_21_H_21_O_9_]^−^), *m*/*z* 418 ([C_21_H_21_O_9_+H]^−^), *m*/*z* 307 ([C_15_H_15_O_7_]^−^), *m*/*z* 335 ([C_16_H_17_O_8_−H]−H^−^), and *m*/*z* 191 ([C_7_H_11_O_6_]^−^).

**Chalcones:** Two chalcones were annotated (compounds **Cha60** and **Cha61**). Compound **Cha60** was annotated as naringenin chalcone through the fragments *m*/*z* 151 ([C_7_H_5_O_4_−H]−H^−^), *m*/*z* 165 ([C_9_H_7_O_3_+2H]^−^), *m*/*z* 119 ([C_8_H_7_O]^−^), *m*/*z* 177 ([C_9_H_7_O_4_−H]−H^−^), and *m*/*z* 228 ([C_14_H_11_O_3_+H]^−^). The naringenin isomer was discarded due to its exact mass (272.25601). Compound **Cha61** was annotated as cardamonin, with fragments at *m*/*z* 134 ([C_8_H_6_O_2_]^−^), *m*/*z* 178 ([C_9_H_6_O_4_]^−^), *m*/*z* 137 ([C_7_H_7_O_3_−H]−H^−^), *m*/*z* 133 ([C_8_H_5_O_2_]^−^), and *m*/*z* 139 ([C_7_H_7_O_3_]^−^), justifying the identification.

**Miscellaneous compounds:** Four additional compounds belonging to diverse chemical classes were annotated. Compound **M62** was annotated as uridine, possessing *m*/*z* 200, *m*/*z* 42, *m*/*z* 153, *m*/*z* 140, and *m*/*z* 71 fragments due to the [C_8_H_11_NO_5_]−H^−^, [CHNO]−H^−^, [C_6_H_6_N_2_O_3_]−H^−^, [C_6_H_8_NO_3_−H]−H^−^, and [C_3_H_3_O_2_]^−^ moieties, respectively. Compound **M63** was annotated as guanosine, presenting the typical fragments of *m*/*z* 150, *m*/*z* 133, *m*/*z* 117, *m*/*z* 113, and *m*/*z* 191 from [C_5_H_4_N_5_O]^−^, [C_5_H_3_N_4_O−H]−H^−^, [C_5_H_2_N_4_]−H^−^, [C_5_H_7_O_3_−H]−H^−^, and [C_7_H_7_N_5_O_2_−H]−H^−^ moieties, respectively. Compound **M64** was annotated as khelloside, possessing the typical fragment of *m*/*z* 246 ([C_13_H_9_O_5_]^−^), corresponding to the hexoside moiety loss [M−H−162].

**Non-annotated compounds:** Twelve metabolites detected in the *V. corymbosum* extracts were not annotated. **Nid1** and **Nid2** are two isomers possessing the structural formula C_5_H_10_O_4_ and exact mass [M−H]^−^ of 133.05058(86), respectively, but were not annotated. Compound **Nid3** has a structural formula (C_6_H_8_O_2_) and an exact mass compatible with a hexadienoic acid, including the well-known sorbic acid. However, its unique fragment of *m*/*z* 69 (despite being present in the mass spectra of hexadienoic acids) prevents its unequivocal identification. Compounds **Nid4** and **Nid5** appear to be two isomers of structural formula C_6_H_10_O_3_ with an exact mass [M−H]^−^ of 129.05580(69), which were not possible to identify. Compound **Nid6** appears to be an acidic compound due to the fragment of *m*/*z* 121, which corresponds to the typical loss of −CO_2_ in carboxylic acids [M−44]^−^. Compounds **Nid7** and **Nid8**, **Nid9** and **Nid10**, and **Nid11** and **Nid12** appear to correspond to three pairs of isomers due to their structural formulas and exact masses; however, it was not possible to identify them. Isomers **Nid9** and **Nid10** have a strong probability of having a glucosyl moiety in their structure due to their typical fragment of *m*/*z* 161.

### 3.2. Metabolome Semi-Quantitative Evaluation

#### 3.2.1. Global Metabolome Cultivars Comparison

**Figure 2:**
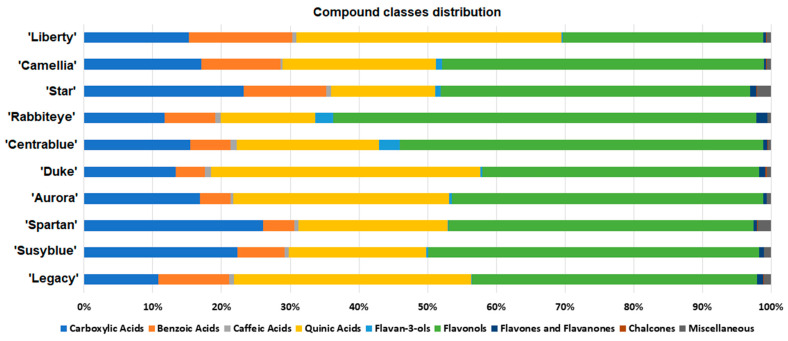
Compounds class distribution in *V. corymbosum* cultivars.

The annotated *V. corymbosum* metabolites were grouped into 10 distinct compound classes, and their relative amounts per cultivar are presented in Figure 2. Carboxylic, benzoic, and quinic acids and flavonols represent the great majority of the annotated metabolites in all cultivars. Carboxylic acids represent nearly 11–26% of the annotated metabolites, with the lowest and the highest quantities observed for the ‘Legacy’ and ‘Spartan’ cultivars, respectively. Benzoic acids represent around 4–15%, with ‘Aurora’ and ‘Liberty’ possessing the lowest and the highest amount of this compound class. Quinic acids vary from just under 13% in ‘Star’ and ‘Rabbiteye’ to nearly 38% in the ‘Liberty’ and ‘Duke’ cultivars. Flavonols range from around 30% of the annotated metabolites in ‘Liberty’ to 60% in ‘Rabbiteye’.

‘Rabbiteye’ and ‘Centrablue’ were the cultivars possessing the highest amount of flavan-3-ols, nearly 3%, while ‘Star’ and ‘Spartan’ were the ones possessing the highest amount of the miscellaneous compounds (uridine, guanosine, and khelloside). Overall, caffeic acids, flavones, flavanones, and chalcones each represent less than 2% of the annotated *V. corymbosum* metabolites.

**Figure 3:**
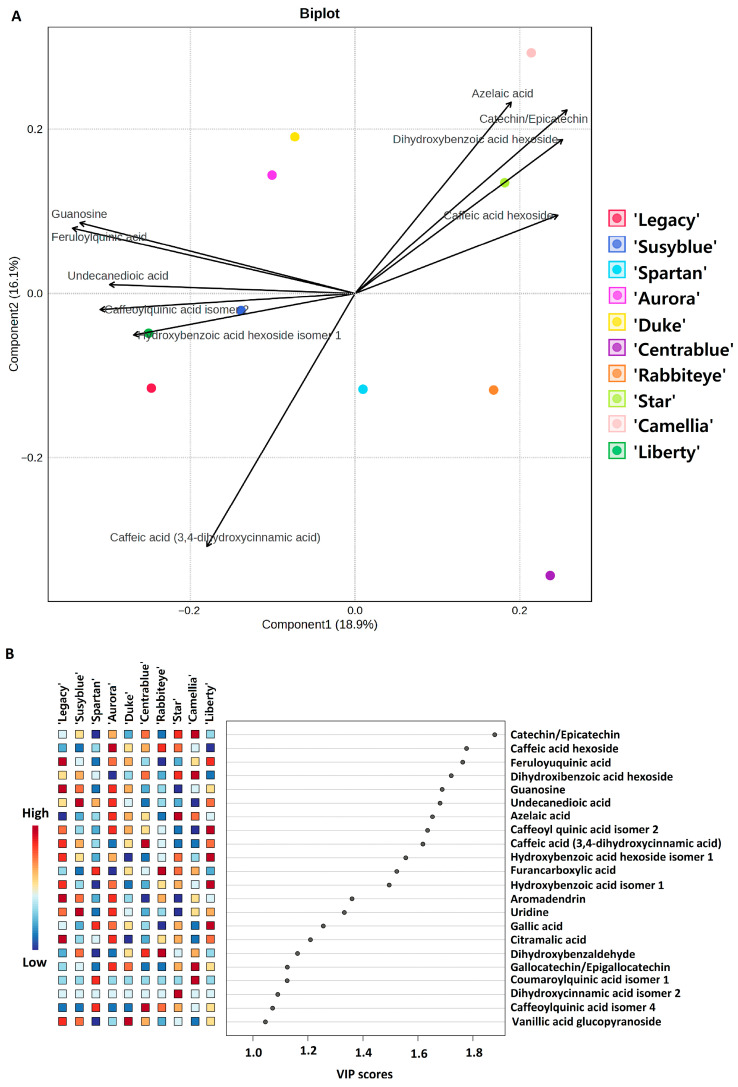
(**A**) PLSDA biplot (scores and loadings—10 most relevant) obtained with the metabolite profile (semi-quantitative data) of *V. corymbosum*, and (**B)** variables’ importance on projection (VIP scores) of the PLSDA model—23 most relevant (VIP > 1).

A PLSDA model and the corresponding VIP scores were obtained from the metabolites’ relative amount in all cultivars (Figure 3A,B) with two main goals: (I) to infer about the similarities/differences among cultivars’ metabolome, and (II) to identify the metabolites that strongly correlate with each cultivar. Figure 3A presents the biplot of the PLSDA models, including the scores of the models and the 10 most relevant metabolites (for simplification proposes) contributing to the cultivars’ distribution across the score map. The PLSDA was cross-validated (CV) through a fivefold CV method (R2—0.99618; Q2—0.98151). In Figure 3B, the 25 most relevant metabolites are presented, including the degree of correlation with each cultivar. The 10 metabolites that differ the most among the 10 analysed *V. corymbosum* cultivars are as follows: azelaic acid; catechin/epicatechin; dihydroxybenzoic acid hexoside; caffeic acid hexoside; caffeic acid; guanosine, feruloylquinic acid; undecanedioic acid; caffeoylquinic acid isomer 2; and hydroxybenzoic acid hexoside isomer 1. The ‘Star’ and ‘Camellia’ cultivars appear in the positive part of both components, suggesting a similar metabolome profile. These two cultivars positively correlate with the amount of azelaic acid, catechin/epicatechin, dihydroxybenzoic acid hexoside, and caffeic acid hexoside and negatively correlate with caffeic acid. The ‘Spartan’, ‘Rabbiteye’, and ‘Centrablue’ cultivars all appear in the positive part of component 1 and the negative part of component 2, pointing to a similar metabolome profile. These three cultivars positively correlate mainly with the relative amount of caffeoylquinic acid isomer 4, dihydroxybenzaldehyde, furancarboxylic acid, and coumaroylquinic acid (only the ‘Spartan’ cultivar), as can be seen in the VIP scores (Figure 3B). Also, they negatively correlate with the amount of guanosine and feruloylquinic acid. The ‘Duke’ and ‘Aurora’ cultivars cluster in the negative part of the first component and the positive part of the second and positively correlate mainly with caffeic acid hexoside, feruloyl quinic acid, guanosine, azelaic acid (‘Aurora’), and with vanillic acid glucopyranoside (‘Duke’). ‘Legacy’, ‘Liberty’, and ‘Susyblue’ appear in the negative part of both components and strongly correlate with the relative amount of caffeoylquinic acid isomer 2, hydroxybenzoic acid hexoside isomer 1, and caffeic acid. These three cultivars negatively correlate with azelaic acid, catechin/epicatechin, dihydroxybenzoic acid hexoside, and caffeic acid hexoside.

Additionally, a non-parametric ANOVA was performed for the 10 most discriminating metabolites (according to the VIP scores). The comparative analysis of the metabolites revealed statistically significant differences for all compounds according the Kruskal–Wallis test. Subsequent pairwise comparisons using the Mann–Whitney U test with Holm correction identified specific differences among cultivars, particularly involving ‘Camellia’, ‘Aurora’, and ‘Star’, which consistently exhibited negligible or null concentrations for several metabolites, in contrast to cultivars such as ‘Liberty’ and ‘Susyblue’, which showed markedly elevated levels (Table 2).

Table 2 Pairwise comparisons of cultivars for each compound showing statistically significant differences in metabolite concentrations. Results are based on Mann–Whitney U tests with Holm correction for multiple comparisons. Differences were considered statistically significant at a corrected *p*-value threshold of *p* < 0.05 

#### 3.2.2. Specific Metabolites’ Variation Between Cultivars

**Figure 4:**
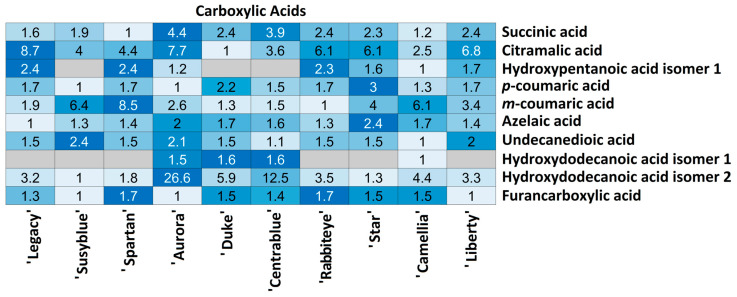
Carboxylic acids’ relative abundance in *V. corymbosum* cultivars.

**Figure 5:**
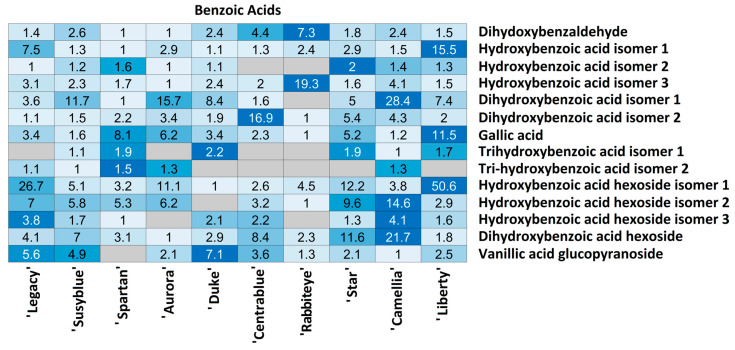
Benzoic acids’ relative abundance in *V. corymbosum* cultivars.

**Figure 6:**
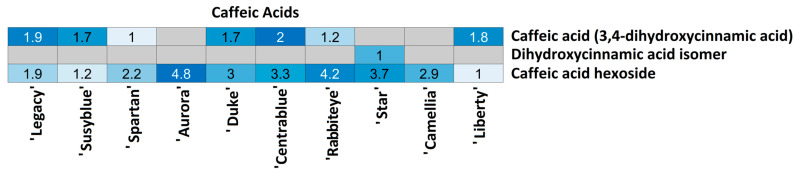
Caffeic acids’ relative abundance in *V. corymbosum* cultivars.

**Figure 7:**
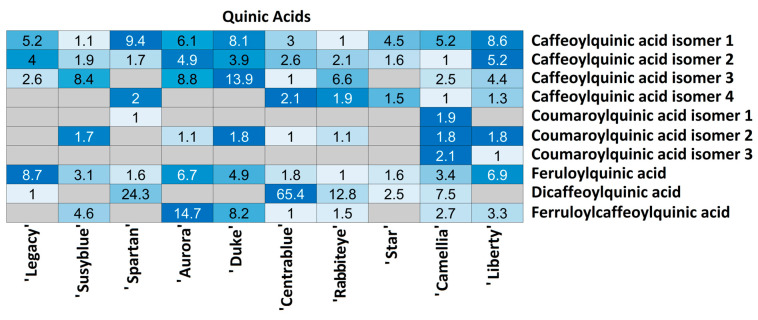
Quinic acids’ relative abundance in *V. corymbosum* cultivars.

**Figure 8:**
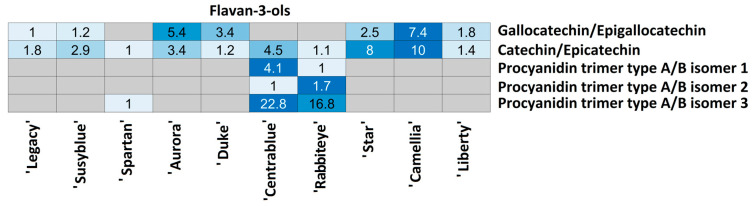
Flavan-3-ols’ relative abundance in *V. corymbosum* cultivars.

**Figure 9:**
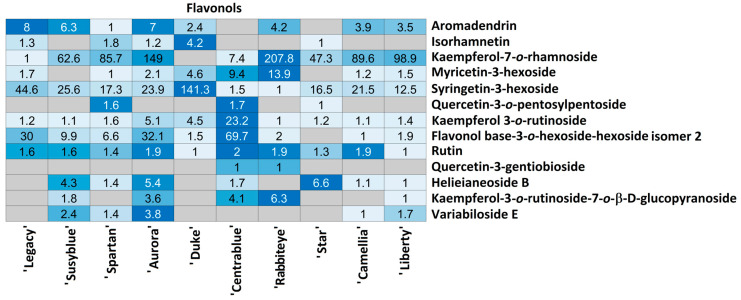
Flavonols’ relative abundance in *V. corymbosum* cultivars.

**Figure 10:**
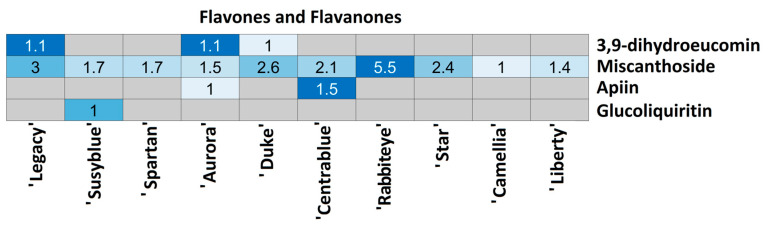
Flavones’ and flavanones’ relative abundance in *V. corymbosum* cultivars.

**Figure 11:**
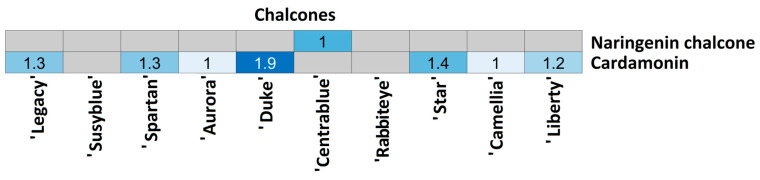
Chalcones’ relative abundance in *V. corymbosum* cultivars.

**Figure 12:**
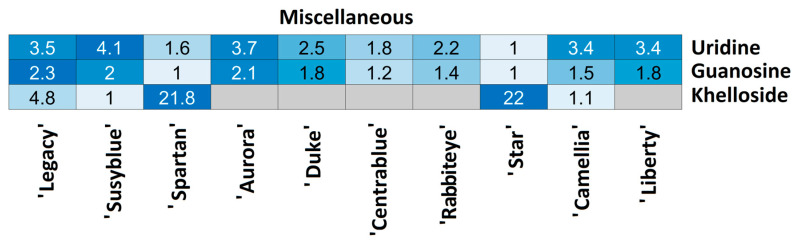
Miscellaneous compounds’ relative abundance in *V. corymbosum* cultivars.

For each compound class, a heatmap was generated (Figure 4, Figure 5, Figure 6, Figure 7, Figure 8, Figure 9, Figure 10, Figure 11 and Figure 12) according to the method described in Section 2.4. Data Analysis. The values inside each cell correspond to the ratio between the corresponding cultivar area and the lowest cultivar area for each metabolite. Grey cells correspond to an undetected metabolite in the corresponding cultivar when the extract ion chromatograms were obtained for each exact mass with a tolerance of ±0.01. It should be stressed that these heatmaps allow metabolite semi-quantitative comparisons between cultivars but not quantitative comparisons among metabolites of a single cultivar.

In total, 10 carboxylic acids were annotated, of which 8 were detected in all cultivars, while 2 of them (hydroxypentanoic isomer 1 and hydroxydodecanoic isomer 1) were undetected in 3/10 and 6/10 cultivars (Figure 4), respectively.

In the ‘Aurora’ and ‘Camellia’ cultivars, all the carboxylic acids were detected. Regarding the relative quantity of each metabolite among the cultivars, quite different variations can be observed. Hydroxypentanoic acid isomer 1, *p*-coumaric acid, azelaic acid, undecanedioic acid, hydroxydodecanoic acid isomer 1, and furancarboxylic acid (6/10) relative quantities do not vary more than 2.5 times between cultivars. However, metabolites such as succinic acid, citramalic acid, and *m*-coumaric acid present inter-cultivar variations of 8.7 times. Hydroxydodecanoic acid isomer 2 is the metabolite presenting the highest variation among cultivars, with the lowest quantity present in the ‘Susyblue’ cultivar and the highest in the ‘Aurora’ cultivar (26.6 times).

It should be emphasised that all the carboxylic acids annotated were detected only in the ‘Camellia’ and ‘Aurora’ cultivars.

Regarding benzoic acids, 14 metabolites were annotated, 7 of which were present in all cultivars, while the remaining 7 were randomly annotated among cultivars (Figure 5).

‘Rabbiteye’ emerges as the cultivar presenting the fewest benzoic acids (9/14), while ‘Susyblue’ and ‘Camellia’ present all the annotated ones. Most of the annotated benzoic acids strongly varied among cultivars. A few exceptions occur for hydroxybenzoic acid isomer 2 and trihydroxybenzoic acid isomers 1 and 2, for which the variations between cultivars are less than or equal to 2.2. Metabolites such as dihydroxybenzaldehyde, hydroxybenzoic acid hexoside isomer 3, and vanillic acid glucopyranoside present inter-cultivar variations of up to 10 times. Higher metabolite variations among cultivars were observed for hydroxybenzoic acid isomer 1 (up to 15.5 times) and isomer 3 (up to 19.3 times), dihydroxybenzoic acid isomer 1 (up to 28.4 times) and isomer 2 (up to 16.9 times), gallic acid (up to 11.5 times), dihydroxybenzoic acid hexoside (up to 21.7 times), and with a considerable variation between cultivars (up to 50.6 times), hydroxybenzoic acid hexoside isomer 1. This benzoic acid strongly fluctuates among cultivars, with the lowest quantity annotated in ‘Duke’ and the highest in ‘Liberty’ (50.6 times higher). ‘Legacy’ and ‘Star’ cultivars also present high amounts of this compound, 26.7 and 12.2 times higher than ‘Duke’. It should be stressed that all the annotated benzoic acids were detected in the ‘Camellia’ and ‘Susyblue’ cultivars.

Three caffeic acids were annotated in *V. corymbosum* cultivars (Figure 6). Caffeic acid was annotated in 7/10 cultivars with quite a similar amount in all cultivars (variations were no higher than 1.9 times). This metabolite was undetected in the ‘Aurora’, ‘Star’, and ‘Camellia’ cultivars. Dihydroxycinnamic acid was only annotated in the ‘Star’ cultivar, preventing additional comparison. Caffeic acid hexoside was annotated in all cultivars, with inter-cultivar variation no higher than 4.8 times. The lowest amounts was measured in ‘Liberty’ and ‘Susyblue’ (1.2 times higher than ‘Liberty’), and the highest amounts were annotated in ‘Rabbiteye’ (4.2 times more than ‘Liberty’) and ‘Aurora’ (4.8 times more than ‘Liberty’).

Quinic acids were randomly detected between cultivars (Figure 7). Ten metabolites were annotated, of which only (3/10) were annotated in all cultivars: caffeoylquinic acid isomers 1 and 2 and feruloylquinic acid. Four isomers of caffeoylquinic acid were annotated: isomers 1 and 2 in all cultivars, with relative amounts between cultivars no higher than 9.4 and 5.2, respectively, and isomers 3 and 4 randomly annotated among cultivars. The lowest relative amount of isomer 3 was detected in ‘Rabbiteye’ and the highest in ‘Duke’ (13.9 times higher), while isomer 4 was detected in the lowest relative amount in ‘Camellia’ and the highest in ‘Centrablue’ (2.1 times higher). Three coumaroylquinic acid isomers were randomly annotated between cultivars in very similar amounts (no more than 2.1 times). ‘Camellia’ was the unique cultivar in which all the isomers were annotated and also presented the highest quantity of all of them, while none of the three were annotated in the ‘Star’ cultivar. Feruloylquinic acid was annotated in all cultivars, with the lowest relative amount observed in ‘Rabbiteye’ and the highest in ‘Legacy’ (8.7 times higher). Dicaffeoylquinic acid was annotated in 5/10 cultivars with a huge variation among them; the lowest relative amount was observed in ‘Legacy’, while the highest was observed in ‘Centrablue’ (65.4 times higher). Feruloylquinic acid was detected in 7/10 cultivars; the lowest amount was observed in ‘Centrablue’, and the highest was observed in ‘Aurora’ (14.7 times higher). It should be stressed that all the annotated quinic acids were detected only in the ‘Camellia’ cultivar.

Five metabolites from the flavan-3-ol class were annotated in the *V. corymbosum* extracts (Figure 8). Gallocatechin/epigallocatechin was observed in 7/10 cultivars, catechin/epicatechin in all cultivars, and three procyanidin trimer type A/B isomers only in the ‘Centrablue’ and ‘Rabbiteye’ cultivars. Isomer 3 was also annotated in the ‘Spartan’ cultivar. ‘Legacy’ possesses the lowest gallocatechin/epigallocatechin relative amount, while ‘Camellia’ has the highest (7.4 times more). This metabolite was undetected in ‘Spartan’, ‘Centrablue’, and ‘Rabbiteye’. A lower amount of catechin/epigallocatechin was detected in ‘Spartan’ and the highest in ‘Camellia’ (10 times higher). ‘Rabbiteye’ possesses the lowest relative quantity of procyanidin isomer 1 and ‘Centrablue’ the highest (4.1 times). Regarding isomer 2, the lowest relative amount was observed in ‘Centrablue’, while the highest was observed in ‘Rabbiteye’ (1.7 times higher). Isomer 3 was observed in the lowest amount in ‘Spartan’, while ‘Rabbiteye’ possesses 16.8 times more and ‘Centrablue’ 22.8 times more.

Flavonols were randomly annotated among the ten cultivars (Figure 9). Rutin was annotated in all cultivars, presenting very little variation among them (no more than 1.9 times). Kaempferol-3-*O*-rutinoside was also annotated in all cultivars; however, it exhibited higher variation: the lowest amount was measured in ‘Rabbiteye’ and the highest in ‘Centrablue’ (23.2 times higher). Quercetin-3-gentibioside was only annotated in ‘Centrablue’ and ‘Rabbiteye’ in the same relative amount. Aromadendrin, isorhamnetin, myricetin-3-hexoside, and quercetin-3-*O*-pentosylpentoside were randomly detected among cultivars, presenting variations no higher than 13.9 times. Among the metabolites that present higher variation among cultivars are kaempferol-7-*O*-rhamnoside (‘Legacy’—lowest; ‘Rabbiteye’—207.8 times more), syrigetin-3-hexoside (‘Rabbiteye’—lowest; ‘Duke’—141.3 times more), and flavonol base-3-*O*-hexoside-hexoside isomer 2 (‘Camellia’—lowest; ‘Centrablue’—69.7 times more). Helieianeoside B, kaempferol-3-*O*-rutinoside-7-*O*-B-D-glucopyranoside, and variabiloside E were not detected in the ‘Legacy’ and ‘Duke’ cultivars. These metabolites were randomly detected in the remaining cultivars, presenting variation among them no higher than 6.6 times.

Three flavanones (3,9-dihydroeucomin, miscanthoside, and glucoliquiritin) and one flavone (apiin) were annotated among *V. corymbosum* cultivars (Figure 10). 3,9-dihydroeucomin was detected only in ‘Legacy’, ‘Aurora’, and ‘Duke’ in the same relative amount and glucoliquiritin only in ‘Susyblue’. Miscanthoside was annotated in all the cultivars, with ‘Camellia’ being the one with the lowest amount and ‘Rabbiteye’ the one with the highest (5.5 times higher). The unique flavone annotated in *V. corymbosum* extracts was only detected in ‘Aurora’ (lowest relative amount) and ‘Centrablue’ (1.5 times higher).

Two chalcones were annotated among *V. corymbosum* cultivars (Figure 11), with naringenin chalcone detected uniquely in ‘Centrablue’. Cardamonin was not detected in ‘Susyblue’, ‘Centrablue’, or ‘Rabbiteye’ and was detected in nearly the same amount in the remaining cultivars. The lowest relative amount was measured in ‘Camellia’, while the highest was in ‘Duke’ (1.9 times higher).

Three additional metabolites were annotated in *V. corymbosum* extracts (Figure 12). Uridine and guanosine were annotated in all cultivars, presenting a slight variation among them. ‘Star’ was the cultivar presenting the lowest quantity of these two metabolites, while the highest quantity was measured in ‘Susyblue’ (4.1 times higher) and ‘Legacy’ (2.3 times higher), respectively. Khelloside was detected in the lowest amount in both the ‘Camellia’ and ‘Susyblue’ cultivars, while also being detected in ‘Legacy’ (4.8 times more) and in ‘Spartan’ and ‘Star’ (nearly 22 times more for both).

## 4. Discussion

In this work, LC-MS/MS was used to decipher the metabolic profile of *V. corymbosum* ethanolic leaf extracts of 10 distinct cultivars (‘Legacy’, ‘Susyblue’, ‘Spartan’, ‘Aurora’, ‘Duke’, ‘Centrablue’, ‘Rabbiteye’, ‘Star’, ‘Camellia’, and ‘Liberty’). Three plants of the ‘Duke’ and ‘Legacy’ cultivars and a single plant of each of the remaining eight cultivars (the only ones provided) were included. Despite the well-known intrinsic plant variability, we have observed for the ‘Duke’ and ‘Legacy’ cultivars a strong metabolite (*m*/*z* values) consistency among the replicates. Replicate variability was only observed in a few *m*/*z* values presenting intensities bellow the defined threshold (1000) for analysis/identification. Considering the above, we believe that the results presented herein are not compromised. A total of 76 metabolites were detected in the extracts, of which 64 were annotated and 12 were non-annotated. The major compound classes were carboxylic, benzoic, caffeic, and quinic acids, flavan-3-ols, flavonols, flavones, flavanones, and chalcones. These compound classes had already been found in the berries and leaves of some *Vaccinium* species, including in bilberries (*Vaccinium vitis*-*idaea*) and blueberries (*V. corymbosum*) [7,9,10,15]. Among the already-published studies, a wide variety of extraction solvents, geographic origins, cultivars, and harvest seasons were found, preventing direct comparisons with the results herein obtained. It is well known that all these factors highly affect plant metabolites. Venskutonis and colleagues [9] obtained *V. corymbosum* leaf extracts with different polarity solvents to obtain diverse polarity fractions and to discuss the impact of the solvent in the extraction and further plant metabolome identification. These authors compared diverse cultivars (not included in this work) and observed that rutin, chlorogenic, and quinic acid concentrations for the same cultivar were highly dependent on the extraction solvent. This solvent-dependent metabolome extraction was also reported in berries [16]. Other authors have used different solvents to extract different compound classes [17]. The harvest season has also been linked to different metabolome several times [7,18]. Despite recognised metabolomic variation between plant cultivars, to the best of our knowledge, no studies have been conducted to compare the metabolome of *V. corymbosum* cultivars’ leaves. Of the more complete exhaustive studies, all but one (Wu et al.) include no more than three cultivars. Wu et al. [10] claimed the analysis of 73 blueberry cultivars from *V. corymbosum* and *V. ashei* species (including ‘Duke’, ‘Legacy’, and ‘Spartan’ included in this work), identifying 23 phenolic compounds. However, these authors grouped all cultivars (in five categories) and presented the overall results per category, preventing specific cultivar characterisation. Eight of the most predominant phenolics were identified in Wu et al.’s study and further quantified, namely five caffeoylquinic acids (3-, 4-, and 5-*O*-caffeoylquinic acid; 3,5- and 4,5-dicaffeoylquinic acid), two quercetin-glycosides (quercetin-3-*O*-glucoside and quercetin-3-*O*-galactoside), and one kaempferol glycoside (kaempferol-3-*O*-glucoside). 3-*O*-caffeoylquinic acid was identified as the predominant compound among the eight quantified in the five categories, followed by quercetin-3-*O*-galactoside. The lowest content overall was obtained for 4-*O*-caffeoylquinic acid. This study annotated four isomers of caffeoylquinic acid (instead of three), but a single dicaffeoylquic acid (instead of two) was annotated. Also, two quercetin- and two kaempferol-based metabolites were annotated, but not the ones reported in Wu et al.’s study. The identification of a higher number of compounds and their correlation with each cultivar is a step forward from the previous knowledge about *V. corymbosum* leaves’ metabolome. In the context of more sustainable agricultural practices, this specific metabolite/cultivar information could be used to guide cultivar selection for cultivation. Akšić et al. [8] also characterised three blueberry cultivar leaves (including ‘Duke’, characterised in this work) and recognised 5-*O*-caffeoylquinic acid and quercetin-3-*O*-galactoside as the major compounds in *V. corymbosum* berries and leaves. These authors also have identified catechin and epigallocatechin, one caffeoylquinic acid (isomer 5-), caffeic acid, one hydroxybenzoic acid (*p*-hydroxybenzoic acid), and *p*-coumaric acid in the ‘Duke’ cultivar. Despite not being able to discriminate among the caffeoylquinic acids isomers detected in the 10 cultivars analysed in our work, isomers 1 and 2 were annotated in all cultivars, suggesting that one of them is 5-*O*-caffeoylquinic acid and the other one is 3-*O*-caffeoylquinic acid. Notably, isomer 2 appeared as one of the ten most relevant metabolites in the PLSDA analysis performed (Figure 4). Also, isomer 5- was identified as the dominant metabolite in *Vaccinium myrtilus* leaves [19], proving its high occurrence in *Vaccinium* species.

Ten carboxylic acids were annotated among the 10 cultivars herein studied; some of them had already been reported in *V. corymbosum*, while others had not. Of note, succinic acid, **C1**, is a compound belonging to the tricarboxylic acid cycle, which is a key metabolic pathway in plants (linked with energy metabolism) and has already been identified in blueberry buds [20] and in *Cicer arietinum* seeds [11]. Citramalic acid, **C2**, is naturally present as an acidic taste component in fruits and has already been identified in apple juice but not in blueberries [21] and *Sesamum indicum* L. cake [12]. Hydroxypentanoic acids, **C3**, are common organic acids already identified in *Solanum lycopersicum* fruits [22], namely the 2-hydroxypentanoic isomer. Coumaric acids (**C4**–**C5**) have been widely identified in plant extracts, including in blueberries [8], *Sesamum indicum* L. cake [12], and *Cicer arietinum* seeds [11]. Azelaic acid has been identified in an Egyptian cultivar of *Sesame indicum* L. cake [11]. Undecanoic acid (**C7**) has already been reported in *Eugenia winzerlinggii* leaves [23] and identified as being involved in plant defence mechanisms. Despite not mentioning hydroxydodecanoic acids (**C8**–**C9**), this study also reported a mixture of other fatty acids involved in the same plant defence mechanism, including tridecanoic and dodecanoic acids. To the best of our knowledge, furancarboxylic acid derivatives had never been annotated in plant materials.

Regarding benzoic acids, compound **B11** was annotated as protocatechuic aldehyde, which is a naturally occurring phenolic compound that can be found in many plants such as grapevine leaves [24] or in the roots of *Taraxacum ohwianum* [25]. Hydroxybenzoic acids (**B12**–**B14**), namely the *para*- isomer, have been widely identified in raspberry and blackberry [26] and in blueberry leaf [8] extracts. However, the *ortho*- and the *meta*- isomers have never been mentioned in blueberries. Di- and trihydroxybenzoic acids (**B15**–**B19**) have also been widely identified in plants, namely gallic acid [12,27]. Hydroxybenzoic acid hexosides (**B20**–**B23**) have been reported in *Helichrysum italicum* extracts [28]. Also, three isomers of vanillic acid hexoside (**B24**) have been identified in sesame cake [12].

Caffeic acids and caffeic acid hexosides (**Caf25**–**Caf27**) are widely identified and well known as plant metabolites, including being present in blueberry leaves [13,28].

Quinic acids are a class of compounds widely distributed among plant material. Several caffeoyl quinic acid isomers (**Q28**–**Q31**) had already been identified in *V. corymbosum* leaves [10], sesame cake [12], and in the bilberry and bog bilberry [27], among others, as reported above. Coumaroyl-, feruloyl-, dicaffeoyl-, and ferruloylcaffeoylquinic acids (**Q32**–**Q37**) have also been identified as plant metabolites being broadly present across species.

With regard to flavan-3-ols, five metabolites were annotated. Catechins (epi) and gallocatechins (epi) (**Fla38**–**Fla39**) have been broadly identified as plant metabolites being present in blueberry leaves and fruits [8], bilberries and bog bilberries [27], and in lingonberries (*Vaccinium vitis*-*idaea* L.) [13]. Procyanidin trimers (type A/B and type B) (**Fla40**–**Fla42**) have been identified in *Vaccinium* species in several studies [13,29,30].

Flavonols are widely recognised as one of the most abundant subclasses of flavonoids in plants, known for their antioxidant, anti-inflammatory, antimicrobial, cardioprotective, and neuroprotective properties. Their occurrence in plant tissues contributes not only to pigmentation but also to stress response and defence mechanisms, such as protection from UV radiation, oxidative stress, and pathogen attack [31]. In this study, thirteen distinct flavonols were annotated. Aromadendrin (**Flo43**) had already been identified in the aerial parts of *Plume armeniaca* [32] but never in *Vaccinium* species. This compound has been associated with a broad spectrum of biological activities, including antioxidant, anti-inflammatory, antimicrobial, and hepatoprotective effects, making its presence in blueberry leaves particularly relevant [33]. Isorhamnetin (**Flo44**) has been identified in *Helichrysum italicum* extracts [28]. Kaempferol-3-*O*-rhamnoside (**Flo45**) has previously been identified in bilberries [27]. Myricetin hexosides (**Flo46)** have been widely identified in several plant species and/or by-products [10,34]. Syringetin-3-hexoside (**Flo47)** has been identified in bilberry and blueberry liqueurs [34] and in bog bilberries (*Vaccinum uliginosum*) but not in bilberries [27]. Quercetin and kaempferol sugar derivatives (**Flo48**–**Flo54**) have been reported as being present in several plant leaves species, including in *V. corymbosum* species, *V. mirtylus* (bilberry and bog bilberry) [27], lingonberry, and hybrid bilberry leaves [13], among others. Variabiloside (**Flo55**) has already been identified in *Strychnos variabilis* leaves [35].

To the best of our knowledge, the flavones and flavanones herein annotated had never been reported in *Vaccinium* species. However, 3,9-dihydroeucomin (**Flav56**) had already been identified in Agave sisalana [36], miscanthoside (**Flav57**) in *Acer truncatum* [37], apiin (**Flav58**) in *Apium graveolens* [38], and glucoliquiritin (**Flav59**) in *Glycyrrhiza uralensis* [39]. Among these, apiin has demonstrated significant anti-inflammatory effects through inhibition of nitric oxide production in activated macrophages [38]. Glucoliquiritin has shown promising antioxidant, anti-inflammatory, and antimicrobial potential in silico, though further in vitro validation is necessary [40]. Khelloside, while not directly tested in isolation, is found in *Ammi visnaga* extracts known for antimicrobial and antioxidant activity [41]. Moreover, khelloside and its aglycone khellin have been described as potent coronary vasodilators and bronchodilators and have also shown hypocholesterolemic effects.

Two chalcones were identified, namely naringenin chalcone (**Cha60**), also present in blueberry fruits [42], and cardamonin (**Cha61**), a chalconoid that has been identified in *Alpina katsumadai* and *Alpina conchigera* [43]. Cardamonin has been extensively studied for its pharmacological properties, including strong anti-inflammatory effects through inhibition of NF-κB and MAPK signalling pathways, as well as notable antioxidant and antimicrobial activities [44]. These bioactivities further support the potential relevance of chalcone-type compounds in *V. corymbosum* leaves for functional and therapeutic applications.

Regarding the three compounds not included in the above-mentioned compound classes, uridine (**M62**) and guanosine (**M63**) have been identified in the seeds of seven distinct Egyptian cultivars of *Cicer arietinum* L. [11], while kelloside (**M64**) has been identified in *Eranthis longistipitata* leaves [45].

Overall, some of the identified metabolites play a key role in disease resistance, environmental adaptability, defence, and stress response in plants. Of note, gallic acid has been reported to be involved in defence response and allelopathic interactions inhibiting pathogen growth and modulating plant–microbial interactions [46]. Rutin and quercetin are linked to enhancing resistance to oxidative stress, UV radiation, and pathogen attack [46]. Azelaic acid is known to prime systemic acquired resistance (SAR) in plants [46]. Catechin/epicatechin and gallocatechin/epigallocatechin are strongly linked to resistance to fungal pathogens (biotic stress) and herbivory insects as well as to abiotic stress [46]. Quinic acids, such as ferruloy-, caffeoyl-, and coumaroyl-, and caffeic acid are also associated with high tolerance to biotic and abiotic stress, with their accumulation often induced under stress conditions [46,47]. *p*-coumaric acid, besides its involvement in plant defence mechanisms, also plays a key role in response to oxidative stress [46,47].

*V. corymbosum* leaves have traditionally been consumed as functional herbal teas, particularly in East Asia and parts of Europe, where they are valued for their antidiabetic, anti-inflammatory, and antioxidant properties [48]. These effects are largely attributed to their rich content of phenolic acids (e.g., caffeic, gallic, and chlorogenic acids) and flavonoids (e.g., quercetin, rutin, and catechins), which are known to scavenge free radicals and modulate oxidative stress pathways [49]. Beyond health benefits, these metabolites also exhibit antimicrobial activity, making them promising candidates as natural food preservatives to enhance shelf life and safety [49]. Recent studies have shown that the total flavonoid content in blueberry leaves can vary significantly among cultivars, influencing their suitability for processing [48]. Cultivars with higher concentrations of flavonols and hydroxycinnamic acids are particularly attractive for extract-based applications in functional beverages, nutraceuticals, and clean-label food products. Industrial pathways may include standardised leaf extract production, encapsulation for controlled release, or integration into active packaging. As consumer demand for plant-based antioxidants grows, blueberry leaves represent a sustainable and underutilised resource with strong potential for value-added food innovation.

Due to its recognised bioactive properties, the identified metabolites in the analysed blueberry ethanolic extracts position this by-product as a good candidate to be used as a functional food in the form of a plant infusion. Despite the fact that most studies have focused on the fruit itself, the available data suggest that the leaves are non-toxic at typical consumption levels, especially when used in infusions or extracts. Indeed, blueberry leaf infusions have traditionally been used in folk medicine to manage blood sugar levels and inflammation. This long-standing use provides some support for their general safety. However, comprehensive safety assessments (e.g., chronic toxicity and genotoxicity) are still lacking and are mandatory for regulatory approval in food applications [50].

## 5. Conclusions

This study presents the most detailed cultivar-specific metabolomic profiling of *V. corymbosum* leaves reported to date. Using UHPLC-QTOF MS, 76 metabolites were detected, of which 64 were confidently annotated and classified into ten major chemical groups. A marked inter-cultivar variability was observed, particularly in the relative abundance of phenolic acids, flavonols, and flavan-3-ols. Multivariate analysis enabled the annotation of discriminant compounds, highlighting the potential of untargeted metabolomics to differentiate cultivars based on their biochemical fingerprints. Several compounds were reported for the first time in *V. corymbosum* leaves, including miscanthoside, glucoliquiritin, apiin, khelloside, aromadendrin, and cardamonin. These metabolites have been associated in other plant sources with relevant bioactivities, such as antioxidant, anti-inflammatory, antimicrobial, vasodilator, and hypocholesterolemic effects. Their presence reinforces the potential of blueberry leaves as a source of bioactive compounds with possible applications in functional foods, nutraceuticals, or cosmetic formulations. These results contribute to the sustainable revalorisation of blueberry leaves, currently considered an agro-industrial residue, and support their integration into bio-based and circular economy strategies. Future work should focus on targeted validation of these bioactivities through in vitro or in vivo assays, as well as the integration of transcriptomic or agronomic data to better understand the regulation of these metabolites. Moreover, extending the comparative metabolomic analysis to other Vaccinium species or to different harvest periods would provide further insights into the genetic and environmental factors underlying the observed variability. Altogether, these findings consolidate the role of *V. corymbosum* leaves as a promising matrix for the development of value-added products.

## Figures and Tables

**Table 1:** Metabolite profiling of *V. corymbosum* ethanolic leaf extracts.

	#	Formula	[M−H]−	Tr	Fragments	mSigma	|Accuracy|(ppm)	Annotation
**Carboxylic Acids**	**C1**	C_4_H_6_O_4_	117.01904	1.98	73.029	4.7	2.48	Succinic acid
	**C2**	C_5_H_8_O_5_	147.03024	1.97	87.009; 85.029; 129.019	7.1	2.38	Citramalic acid
	**C3**	C_5_H_10_O_3_	117.05536	2.95	73.029; 59.014	21.0	3.08	Hydroxypentanoic acid isomer 1
	**C4**	C_9_H_8_O_3_	163.04052	4.95	119.050; 71.014	6.9	3.01	*p*-coumaric acid
	**C5**		163.04048	10.28	119.050	11.8	2.45	*m*-coumaric acid
	**C6**	C_9_H_16_O_4_	187.09791	13.31	125.097; 169.089; 123.082; 143.108	9.4	1.92	Azelaic acid
	**C7**	C_11_H_20_O_4_	215.12888	18.03	197.119; 153.129	17.2	0.01	Undecanedioic acid
	**C8**	C_12_H_24_O_3_	215.16519	17.40	169.157; 87.008; 171.103	8.3	−0.93	Hydroxydodecanoic acid isomer 1
	**C9**		215.16526	19.21	169.160; 197.055; 199.133	8.4	0.88	Hydroxydodecanoic acid isomer 2
	**C10**	C_14_H_22_O_4_	253.14435	19.69	209.155; 210.159; 59.015; 209.120; 89.027	13.2	1.34	Furancarboxylic acid
**Benzoic Acids**	**B11**	C_7_H_6_O_3_	137.02484	1.74	93.038; 111.008; 81.034	4.98	3.07	Dihydoxybenzaldehyde
	**B12**		137.02475	2.86	93.035; 94.038	7.86	2.26	Hydroxybenzoic acid isomer 1
	**B13**		137.02479	11.99	93.035; 108.020	>30	2.70	Hydroxybenzoic acid isomer 2
	**B14**		137.02479	13.04	93.035; 108.022; 94.038	>30	2.70	Hydroxybenzoic acid isomer 3
	**B15**	C_7_H_6_O_4_	153.01959	7.39	109.030; 108.022; 123.046	10.99	2.03	Dihydroxybenzoic acid isomer 1
	**B16**		153.01952	9.82	109.030	>30	1.24	Dihydroxybenzoic acid isomer 2
	**B17**	C_7_H_6_O_5_	169.01453	2.51	125.025	>30	1.66	Gallic acid
	**B18**		169.01441	3.44	125.025; 124.017	>30	0.95	Trihydroxybenzoic acid isomer 1
	**B19**		169.01450	4.20	151.004; 125.024; 83.012	>30	1.48	Tri-hydroxybenzoic acid isomer 2
	**B20**	C_13_H_16_O_8_	299.07716	2.86	137.025; 93.034; 139.040	15.17	0.50	Hydroxybenzoic acid hexoside isomer 1
	**B21**		299.07681	4.05	137.025; 179.036; 239.057; 151.041	16.62	1.17	Hydroxybenzoic acid hexoside isomer 2
	**B22**		299.07666	5.64	137.024; 93.033	>30	1.94	Hydroxybenzoic acid hexoside isomer 3
	**B23**	C_13_H_16_O_9_	315.07196	3.58	152.012; 108.022	16.92	0.63	Dihydroxybenzoic acid hexoside
	**B24**	C_14_H_18_O_9_	329.08707	8.45	167.036; 191.035; 209.047; 123.045	3.37	0.36	Vanillic acid glucopyranoside
**Caffeic Acids**	**Caf25**	C_9_H_8_O_4_	179.03527	8.63	135.046; 134.038	>30	1.62	Caffeic acid (3,4-dihydroxycinnamic acid)
	**Caf26**		179.03529	13.63	135.047; 137.060	>30	1.56	Dihydroxycinnamic acid isomer 2
	**Caf27**	C_15_H_18_O_9_	341.08777	6.20	179.035; 135.035; 181.050	18.7	1.88	Caffeic acid hexoside
**Quinic Acids**	**Q28**	C_16_H_18_O_9_	353.08767	1.39	191.057; 179.036; 135.045; 173.046	15.7	0.20	Caffeoylquinic acid isomer 1
	**Q29**		353.08695	9.09	191.057	27.6	2.44	Caffeoylquinic acid isomer 2
	**Q30**		353.08689	12.65	191.057; 179.035; 135.046; 192.059; 165.018; 173.046	6.0	2.66	Caffeoylquinic acid isomer 3
	**Q31**		353.08662	13.61	191.057; 173.046; 179.036; 135.045	26.2	3.37	Caffeoylquinic acid isomer 4
	**Q32**	C_16_H_18_O_8_	337.09237	10.59	191.057; 173.046	18.1	1.57	Coumaroylquinic acid isomer 1
	**Q33**		337.09225	14.27	163.041; 191.057; 119.051	4.0	1.51	Coumaroylquinic acid isomer 2
	**Q34**		337.09249	16.12	163.041; 191.057; 173.047; 164.044; 119.050	21.5	1.54	Coumaroylquinic acid isomer 3
	**Q35**	C_17_H_20_O_9_	367.10304	9.14	191.057; 173.046; 193.053; 93.035	20.3	0.57	Feruloylquinic acid
	**Q36**	C_25_H_24_O_12_	515.11837	15.48	353.089; 173.046; 179.036; 191.057	1.2	2.19	Dicaffeoylquinic acid
	**Q37**	C_26_H_26_O_12_	529.13358	14.90	367.104; 193.051; 191.056; 353.088; 173.046; 179.037	10.7	3.27	Feruloylcaffeoylquinic acid
**Flavan-3-ols**	**Fla38**	C_15_H_14_O_7_	305.06639	3.88	125.025; 165.020; 167.036; 137.025	16.7	0.43	Gallocatechin/Epigallocatechin
	**Fla39**	C_15_H_14_O_6_	289.07128	5.81	245.082; 203.071; 205.051; 137.025; 179.036	21.8	1.45	Catechin/Epicatechin
	**Fla40**	C_45_H_36_O_18_	863.18313	5.04	411.073; 289.072; 285.041;712.137; 451.104	>30	0.28	Procyanidin trimer type A/B isomer 1
	**Fla41**		863.18424	7.95	289.072; 411.072; 451.104; 711.136; 573.104	25.8	1.15	Procyanidin trimer type A/B isomer 2
	**Fla42**		863.18285	8.58	411.073; 289.073; 451.104; 711.134; 573.103	>30	0.30	Procyanidin trimer type A/B isomer 3
**Flavonols**	**Flo43**	C_15_H_12_O_6_	287.05553	13.52	259.060; 243.069; 125.024; 152.010	24.2	1.43	Aromadendrin
	**Flo44**	C_16_H_12_O_7_	315.05058	18.35	300.028; 301.032; 151.004	16.9	1.46	Isorhamnetin
	**Flo45**	C_21_H_20_O_10_	431.09760	14.60	285.040; 284.033; 191.056	22.1	1.79	Kaempferol-7-*O*-rhamnoside
	**Flo46**	C_21_H_20_O_13_	479.08169	11.82	317.029; 191.056; 301.033	>30	1.42	Myricetin-3-hexoside
	**Flo47**	C_23_H_24_O_13_	507.11269	13.11	344.054	18.5	1.77	Syringetin-3-hexoside
	**Flo48**	C_25_H_26_O_15_	565.11834	11.34	300.027; 463.087; 191.056	>30	3.11	Quercetin-3-*O*-pentosylpentoside
	**Flo49**	C_27_H_30_O_15_	593.14989	11.81	284.033; 463.088; 327.050	13.9	2.24	kaempferol 3-*O*-rutinoside
	**Flo50**		593.15105	12.38	285.042; 284.034	24.8	0.42	Flavonol base-3-*O*-hexoside-hexoside isomer 2
	**Flo51**	C_27_H_30_O_16_	609.14667	11.00	300.029	>30	0.72	Rutin
	**Flo52**	C_27_H_30_O_17_	625.13923	13.20	445.077; 463.088; 301.035; 464.090; 300.029	>30	2.86	Quercetin-3-gentiobioside
	**Flo53**	C_32_H_38_O_20_	741.18837	9.60	300.028; 191.057; 353.087; 417.123; 178.998	>30	0.05	Helieianeoside B
	**Flo54**	C_33_H_40_O_20_	755.20502	12.40	593.152; 285.042	>30	1.40	Kaempferol-3-*O*-rutinoside-7-*O*-β-D-glucopyranoside
	**Flo55**	C_36_H_36_O_18_	755.18240	16.96	593.130; 285.040	77.9	0.04	Variabiloside E
**Flavones and Flavanones**	**Flav56**	C_17_H_16_O_5_	299.09344	18.24	179.035; 151.004; 135.046; 165.020; 121.030; 229.053; 149.063	15.7	3.81	3,9-dihydroeucomin
	**Flav57**	C_21_H_22_O_11_	449.10701	13.53	287.056; 191.057; 151.004; 257.082; 301.033; 135.045	29.9	4.14	Miscanthoside
	**Flav58**	C_26_H_28_O_14_	563.13989	7.74	191.057; 353.088	>30	1.31	Apiin
	**Flav59**	C_27_H_32_O_14_	579.17045	8.66	417.118; 418.123; 307.083; 335.077; 191.057	>30	2.56	Glucoliquiritin
**Chalcones**	**Cha60**	C_15_H_12_O_5_	271.06069	13.03	151.003; 165.055; 119.050; 177.019; 228.076	>30	1.88	Naringenin chalcone
	**Cha61**	C_16_H_14_O_4_	269.08231	19.83	134.037; 178.028; 137.025; 133.029; 139.042	13.9	1.26	Cardamonin
**Miscellaneous**	**M62**	C_9_H_12_N_2_O_6_	243.06211	1.80	200.055; 42.000; 153.029; 140.035; 71.013	10.1	0.45	Uridine
	**M63**	C_10_H_13_N_5_O_5_	282.08430	2.00	150.043; 133.018; 117.019; 113.025; 191.055	10.8	0.21	Guanosine
	**M64**	C_19_H_20_O_10_	407.09745	5.77	245.045	12.0	−2.65	Khelloside

**Table 2:** ANOVA of the ten most discriminating metabolites.

Compound	Cultivar 1	Cultivar 2	Corrected *p*-Value (Holm)
Azelaic acid	‘Aurora’	‘Susyblue’	0.0042
Azelaic acid	‘Aurora’	‘Liberty’	0.0042
Azelaic acid	‘Camellia’	‘Susyblue’	0.0042
Azelaic acid	‘Camellia’	‘Liberty’	0.0042
Azelaic acid	‘Star’	‘Susyblue’	0.0042
Azelaic acid	‘Star’	‘Liberty’	0.0042
Undecanedioic acid	‘Camellia’	‘Susyblue’	0.0042
Undecanedioic acid	‘Camellia’	‘Liberty’	0.0042
Hydroxybenzoic acid hexoside isomer 1	‘Camellia’	‘Liberty’	0.0042
Dihydroxybenzoic acid hexoside	‘Camellia’	‘Liberty’	0.0042
Caffeic acid (3,4-dihydroxycinnamic acid)	‘Aurora’	‘Susyblue’	0.0042
Caffeic acid (3,4-dihydroxycinnamic acid)	‘Aurora’	‘Liberty’	0.0042
Caffeic acid (3,4-dihydroxycinnamic acid)	‘Camellia’	‘Susyblue’	0.0042
Caffeic acid (3,4-dihydroxycinnamic acid)	‘Camellia’	‘Liberty’	0.0042
Caffeic acid (3,4-dihydroxycinnamic acid)	‘Star’	‘Susyblue’	0.0042
Caffeic acid (3,4-dihydroxycinnamic acid)	‘Star’	‘Liberty’	0.0042
Caffeic acid hexoside	‘Camellia’	‘Liberty’	0.0042
Caffeoylquinic acid isomer 2	‘Camellia’	‘Liberty’	0.0042
Feruloylquinic acid	‘Camellia’	‘Liberty’	0.0042
Catechin/Epicatechin	‘Camellia’	‘Liberty’	0.0042
Guanosine	‘Camellia’	‘Liberty’	0.0042

## Data Availability

The datasets presented in this article are not readily available because the data are part of an ongoing study. Requests to access the datasets should be directed to the corresponding author.

## Supplementary Materials

The following supporting information can be downloaded at: https://www.mdpi.com/article/10.3390/foods14162846/s1. Table S1. Structural formula of the fragments (*m*/*z*) experimentally obtained for the annotated *V. corymbosum* metabolites. Table S2. Non-identified metabolites detected in *V. corymbosum* ethanolic leaf extracts.
