# Supplementary material for: Metabolomic Profile of Vaccinium corymbosum Leaves: Exploiting Diversity Among Ten Different Cultivars
Source: Foods. 2025 Aug 17;14(16):2846. doi: 10.3390/foods14162846 (PMC12385233; doi:10.3390/foods14162846)
Supplement: Supplementary file 1 [file foods-14-02846-s001.zip › foods-3733113-supplementary.pdf]

# Supplementary Material

**Metabolomic profile of *Vaccinium corymbosum* leaves: exploiting diversity among ten different cultivars.**

Tânia Ribeiro[1], Manuela Pintado[1]; Clara Sousa[1,*]

Universidade Católica Portuguesa, CBQF – Centro de Biotecnologia e Química Fina – Laboratório Associado, Escola Superior de Biotecnologia, Rua Diogo Botelho 1327, 4169-005 Porto, Portugal

* corresponding author: cssousa@ucp.pt

**Table S1.** Structural formula of the fragments (*m/z*) experimentally obtained for the annotated *V. corymbosum* metabolites.

| | # | Formula | Experimental fragments (*m/z*)[1,2] |
|---|---|---|---|
| Carboxylic Acids | **C1** | $C_4H_6O_4$ | 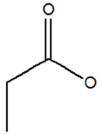 m/z 73 |
| | **C2** | $C_5H_8O_5$ | 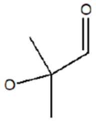 m/z 85 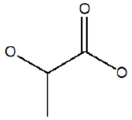 m/z 87 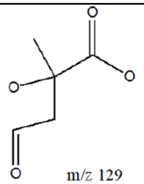 m/z 129 |
| | **C3** | $C_5H_{10}O_3$ | 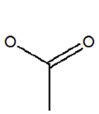 m/z 59 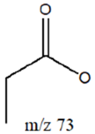 m/z 73 |
| | **C4-C5** | $C_9H_8O_3$ | 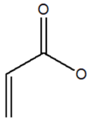 m/z 71 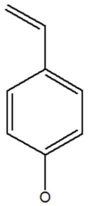 m/z 119 |

| | # | Formula | Experimental fragments (*m/z*)[1,2] |
|---|---|---|---|
| | **C6** | $C_9H_{16}O_4$ | 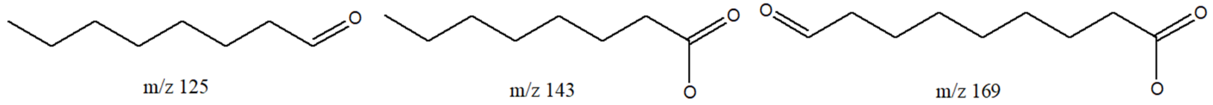 |
| | **C7** | $C_{11}H_{20}O_4$ | 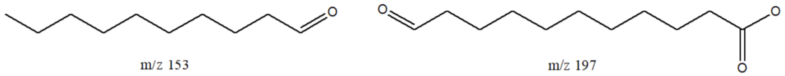 |
| | **C8-C9** | $C_{12}H_{24}O_3$ | 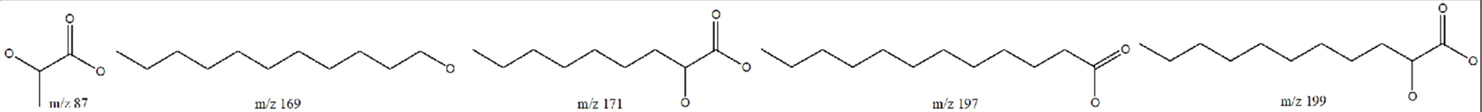 |
| | **C10** | $C_{14}H_{22}O_4$ | 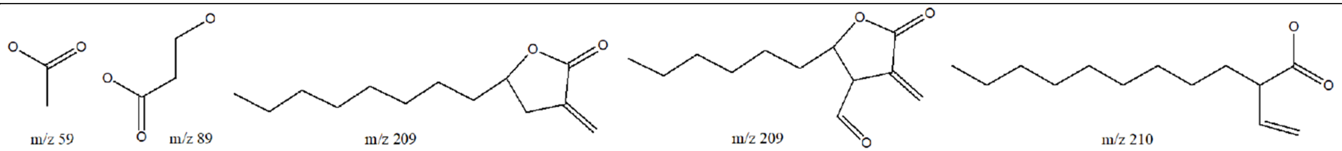 |
| **Benzoic Acids** | **B11-B14** | $C_7H_6O_3$ | 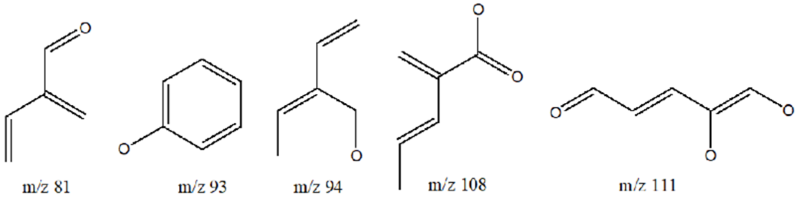 |

| | # | Formula | Experimental fragments (*m/z*)[1,2] |
|---|---|---|---|
| | **B15-B16** | $C_7H_6O_4$ | 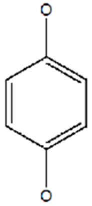 m/z 108 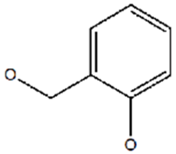 m/z 123 |
| | **B17-B19** | $C_7H_6O_5$ | 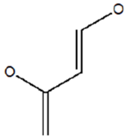 m/z 83 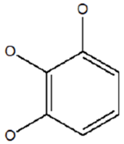 m/z 124 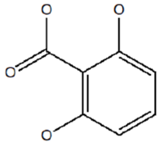 m/z 151 |
| | **B20-B22** | $C_{13}H_{16}O_8$ | 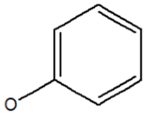 m/z 93 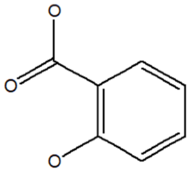 m/z 137 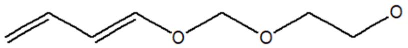 m/z 139 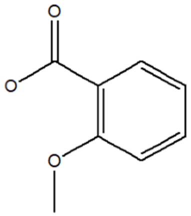 m/z 151 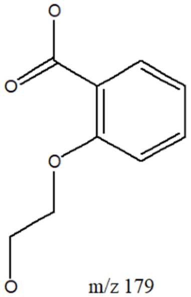 m/z 179 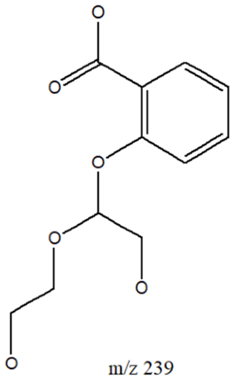 m/z 239 |

| | # | Formula | Experimental fragments (*m/z*)[1,2] |
|---|---|---|---|
| | **B23** | $C_{13}H_{16}O_9$ | 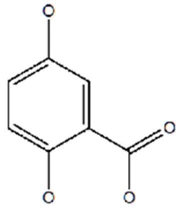 m/z 152 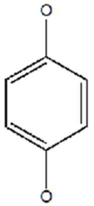 m/z 108 |
| | **B24** | $C_{14}H_{18}O_9$ | 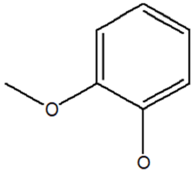 m/z 123 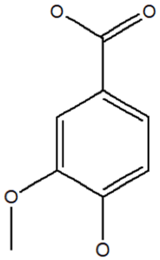 m/z 167 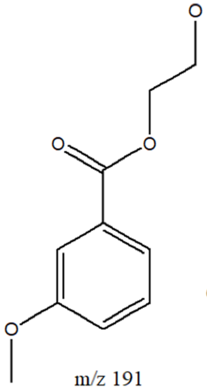 m/z 191 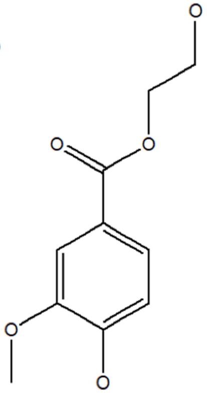 m/z 209 |
| Caffeic Acids | **Caf25-Caf26** | $C_9H_8O_4$ | 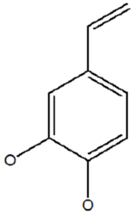 m/z 134 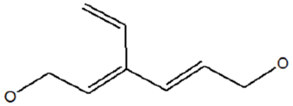 m/z 137 |

| | # | Formula | Experimental fragments (*m/z*)[1,2] |
|---|---|---|---|
| | **Caf27** | $C_{15}H_{18}O_9$ | 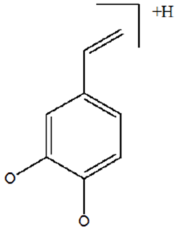 m/z 135 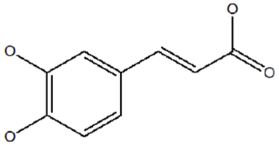 m/z 179 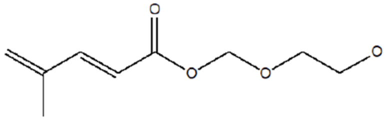 m/z 181 |
| Quinic Acids | **Q28-Q31** | $C_{16}H_{18}O_9$ | 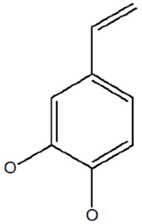 m/z 135 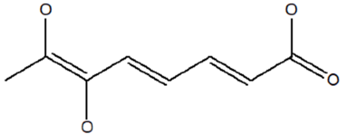 m/z 165 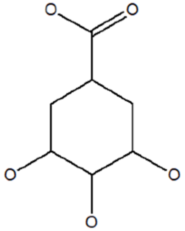 m/z 173 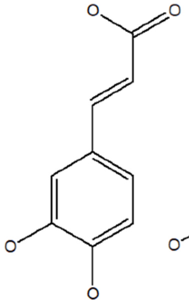 m/z 179 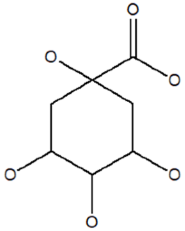 m/z 191 |
| | **Q32-Q34** | $C_{16}H_{18}O_8$ | 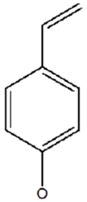 m/z 119 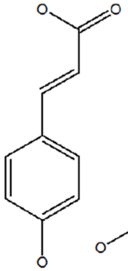 m/z 163 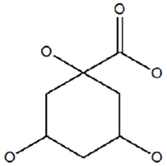 m/z 173 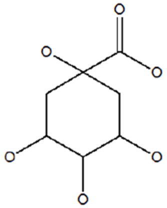 m/z 191 |

| | # | Formula | Experimental fragments (*m/z*)[1,2] |
|---|---|---|---|
| | **Q35** | $C_{17}H_{20}O_9$ | 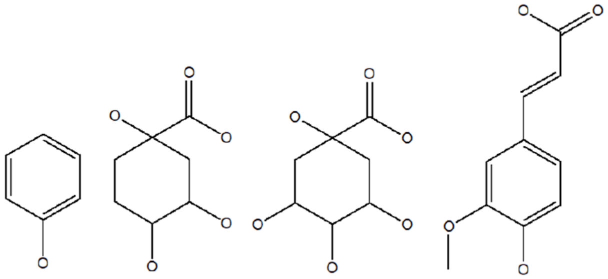 m/z 93 m/z 173 m/z 191 m/z 193 |
| | **Q36** | $C_{25}H_{24}O_{12}$ | 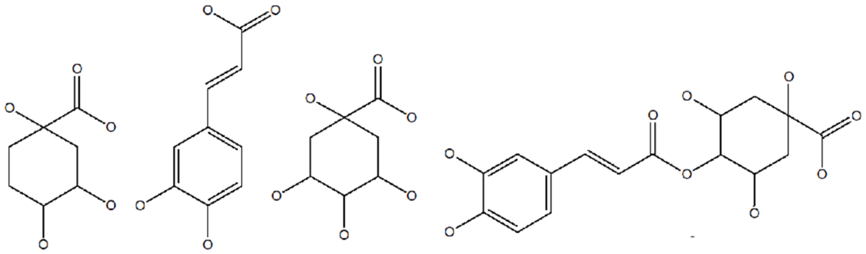 m/z 173 m/z 179 m/z 191 m/z 353 |
| | **Q37** | $C_{26}H_{26}O_{12}$ | 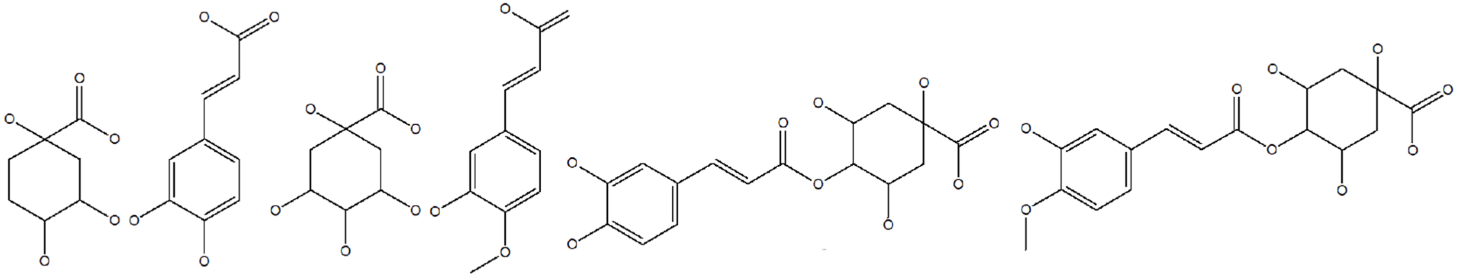 m/z 173 m/z 179 m/z 191 m/z 193 m/z 353 m/z 367 |

| | # | Formula | Experimental fragments ($m/z$)[1,2] |
|---|---|---|---|
| Flavan-3-ols | **Fla38** | $C_{15}H_{14}O_7$ | 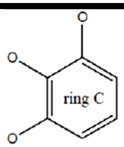 m/z 125 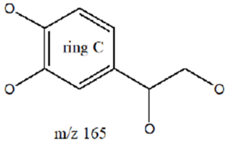 m/z 165 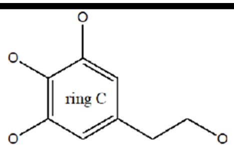 m/z 167 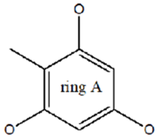 m/z 137 |
| | **Fla39** | $C_{15}H_{14}O_6$ | 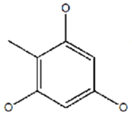 m/z 137 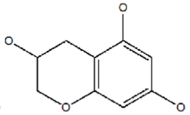 m/z 179 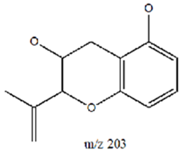 m/z 203 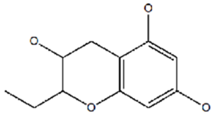 m/z 205 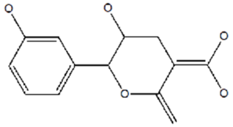 m/z 245 |
| | **Fla40-Fla42** | $C_{45}H_{36}O_{18}$ | 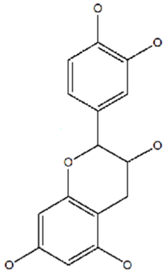 m/z 289 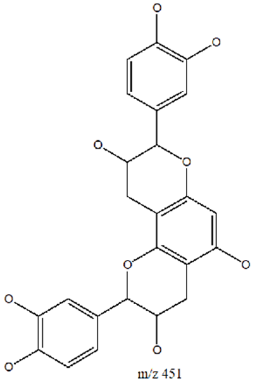 m/z 451 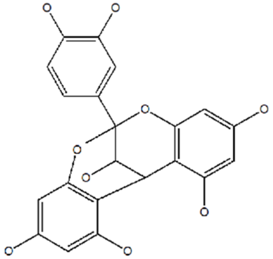 m/z 411 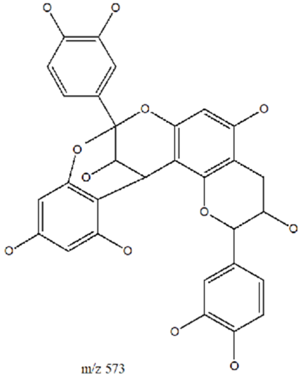 m/z 573 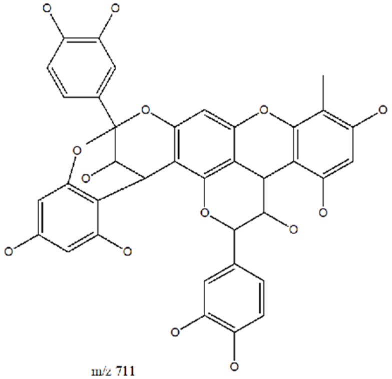 m/z 711 |

| | # | Formula | Experimental fragments (*m/z*)[1,2] |
|---|---|---|---|
| Flavonols | **Flo43** | $C_{15}H_{12}O_6$ | 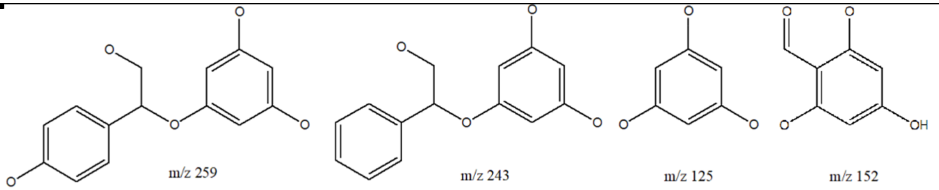 |
| | **Flo44** | $C_{16}H_{12}O_7$ | 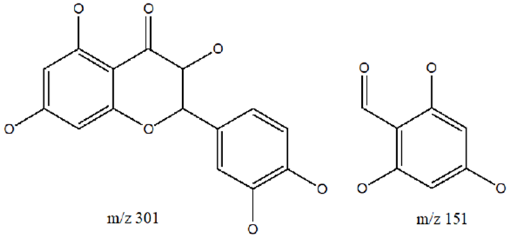 |
| | **Flo45** | $C_{21}H_{20}O_{10}$ | 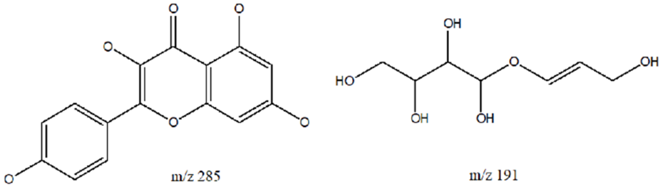 |
| | **Flo46** | $C_{21}H_{20}O_{13}$ | 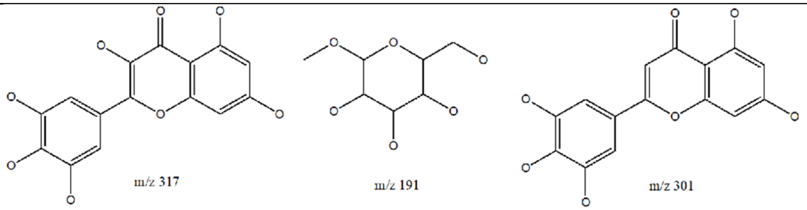 |

| | # | Formula | Experimental fragments (*m/z*)[1,2] |
|---|---|---|---|
| | **Flo47** | $C_{23}H_{24}O_{13}$ | 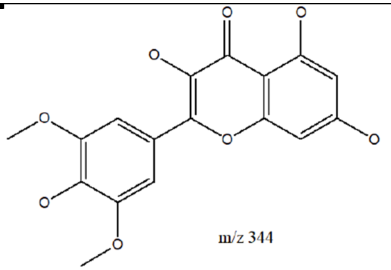 m/z 344 |
| | **Flo48** | $C_{25}H_{26}O_{15}$ | 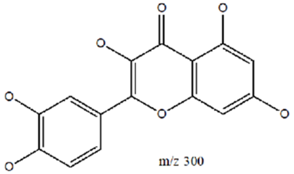 m/z 300 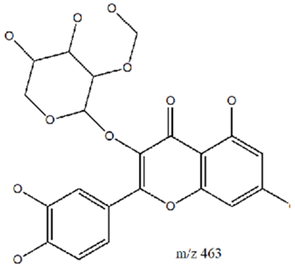 m/z 463 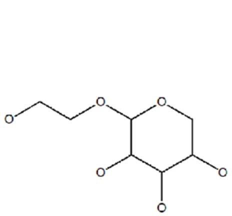 m/z 191 |
| | **Flo49-Flo50** | $C_{27}H_{30}O_{15}$ | 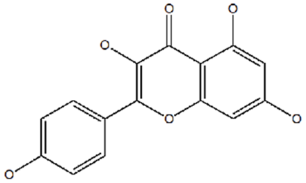 m/z 285 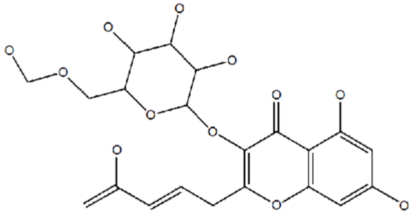 m/z 463 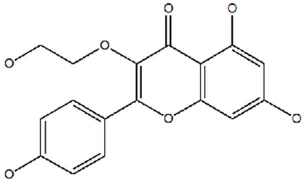 m/z 327 |

| | # | Formula | Experimental fragments (*m/z*)[1,2] |
|---|---|---|---|
| | **Flo51** | $C_{27}H_{30}O_{16}$ | 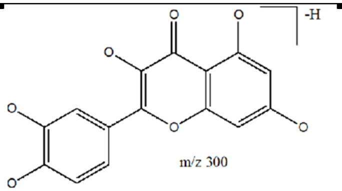 m/z 300 |
| | **Flo52** | $C_{27}H_{30}O_{17}$ | 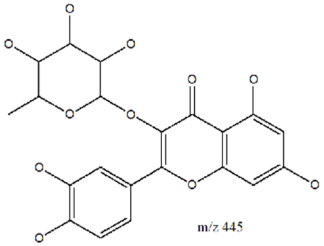 m/z 445 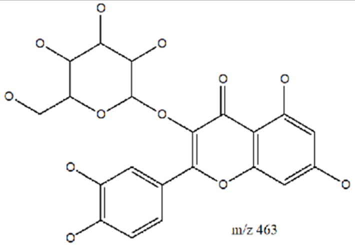 m/z 463 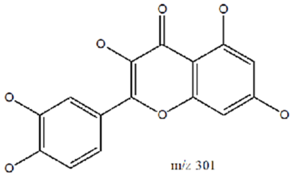 m/z 301 |
| | **Flo53** | $C_{32}H_{38}O_{20}$ | 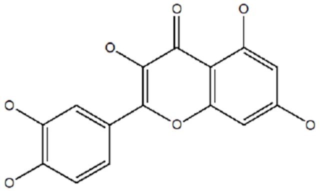 m/z 300 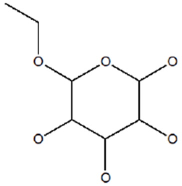 m/z 191 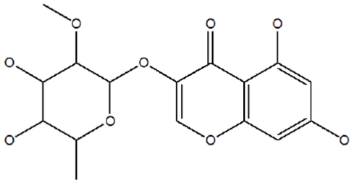 m/z 353 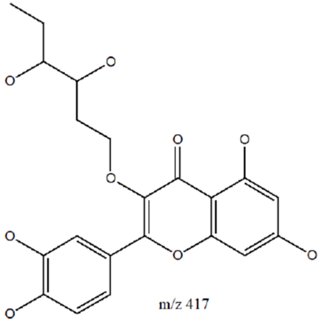 m/z 417 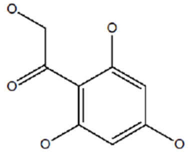 m/z 178 |

| | # | Formula | Experimental fragments (*m/z*)[1,2] |
|---|---|---|---|
| | **Flo54** | $C_{33}H_{40}O_{20}$ | 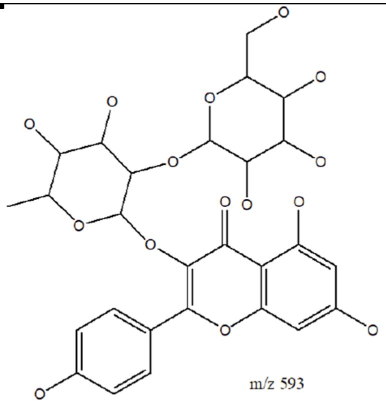<br>m/z 593<br>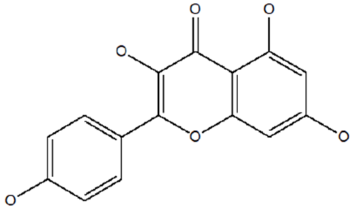<br>m/z 285 |
| | **Flo55** | $C_{36}H_{36}O_{18}$ | 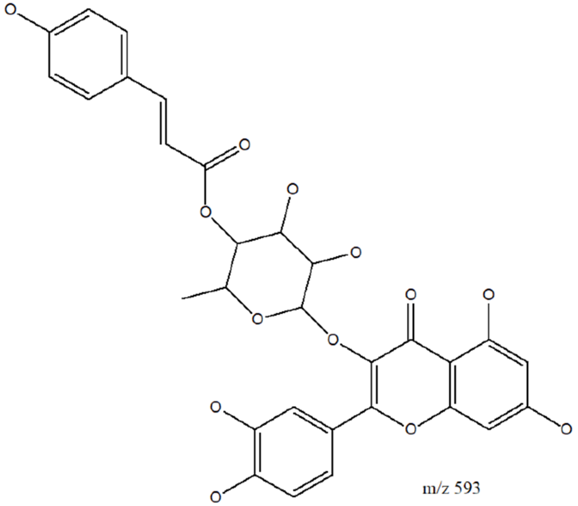<br>m/z 593<br>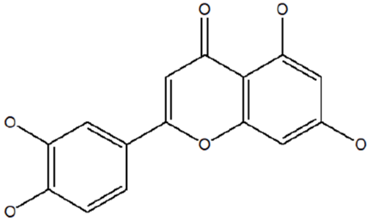<br>m/z 285 |

| | # | Formula | Experimental fragments ($m/z$)[1,2] |
|---|---|---|---|
| Flavones and Flavanones | **Flav56** | $C_{17}H_{16}O_5$ | 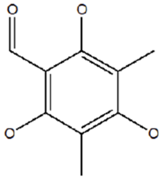 m/z 179 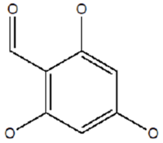 m/z 151 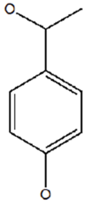 m/z 135 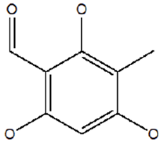 m/z 165 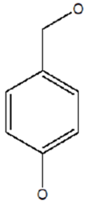 m/z 121 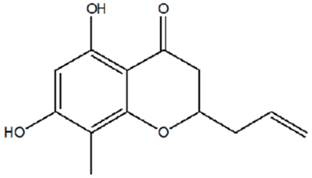 m/z 229 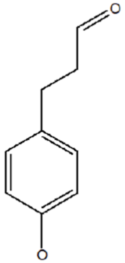 m/z 149 |
| | **Flav57** | $C_{21}H_{22}O_{11}$ | 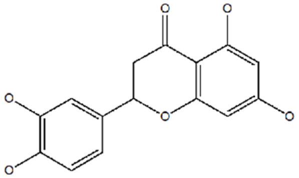 m/z 287 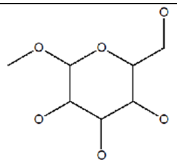 m/z 191 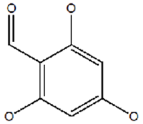 m/z 151 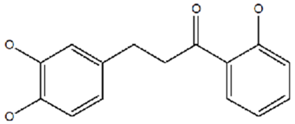 m/z 257 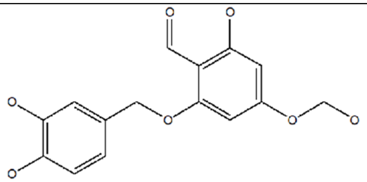 m/z 301 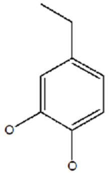 m/z 135 |
| | **Flav58** | $C_{26}H_{28}O_{14}$ | 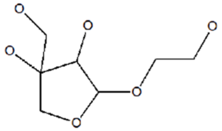 m/z 191 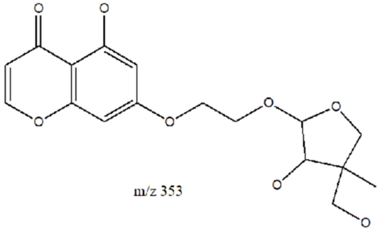 m/z 353 |

| | # | Formula | Experimental fragments (*m/z*)[1,2] |
|---|---|---|---|
| | **Flav59** | $C_{27}H_{32}O_{14}$ | 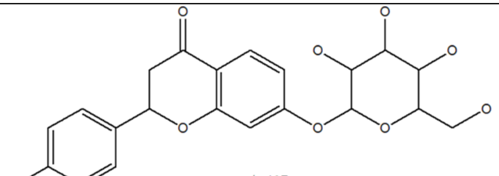 m/z 417 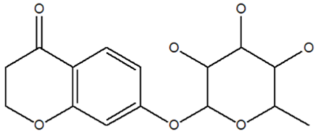 m/z 307 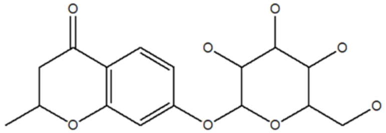 m/z 335 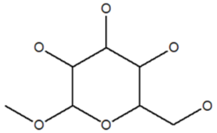 m/z 191 |
| Chalcones | **Cha60** | $C_{15}H_{12}O_{5}$ | 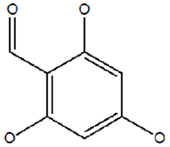 m/z 151 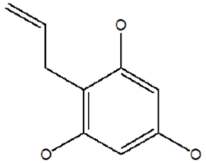 m/z 165 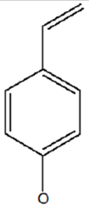 m/z 119 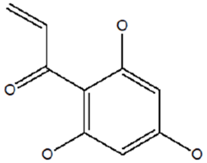 m/z 177 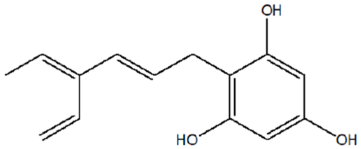 m/z 228 |
| | **Cha61** | $C_{16}H_{14}O_{4}$ | 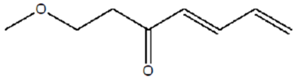 m/z 134 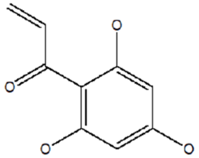 m/z 178 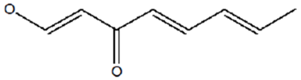 m/z 133 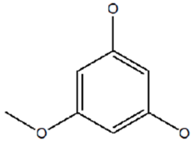 m/z 139 |

|  | # | Formula | Experimental fragments (*m/z*)[1,2] |
|---|---|---|---|
| Miscellaneous | **M62** | $C_9H_{12}N_2O_6$ | 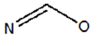 m/z 42 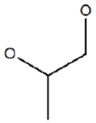 m/z 71 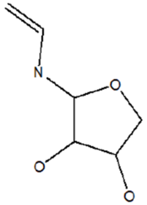 m/z 140 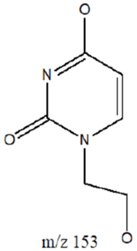 m/z 153 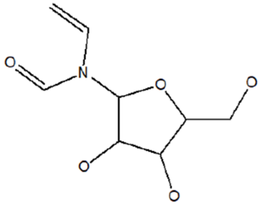 m/z 200 |
|  | **M63** | $C_{10}H_{13}N_5O_5$ | 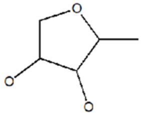 m/z 113 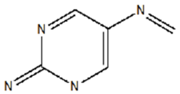 m/z 117 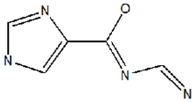 m/z 133 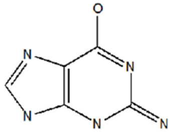 m/z 150 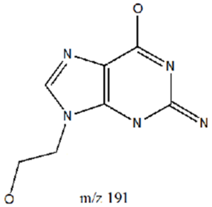 m/z 191 |
|  | **M64** | $C_{19}H_{20}O_{10}$ | 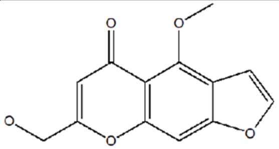 m/z 245 |

[1] In the case of isomers, the position of the oxygen atoms may vary according to the considered one. The presented structures were randomly designed for a single isomer for illustrative proposes.

[2] In the case of fragments whose variations are uniquely related with the presence/absence of a single hydrogen atom, only one fragment was designed for simplification proposes.

**Table S2.** Non-identifyed metabolites detected in *V. corymbosum* ethanolic leaves extracts.

| # | Formula | [M-H]- | tr | Fragments | mSigma | \|Accuracy\|(ppm) |
|---|---|---|---|---|---|---|
| **Nid1** | $C_5H_{10}O_4$ | 133.05058 | 1.84 | 71.014; 72.993; 75.009; 89.025; 59.999 | 12.2 | 0.75 |
| **Nid2** | | 133.05086 | 6.24 | 75.009; 71.014; 72.993; 89.024 | 7.9 | 1.73 |
| **Nid3** | $C_6H_8O_2$ | 111.04471 | 8.77 | 68.995 | 19.2 | 0.27 |
| **Nid4** | $C_6H_{10}O_3$ | 129.05580 | 10.35 | 44.998; 45.026 | >30 | 0.62 |
| **Nid5** | | 129.05569 | 13.84 | 85.029; 44.998; 85.030; 44.999 | >30 | 0.23 |
| **Nid6** | $C_8H_6O_4$ | 165.01951 | 7.11 | 121.029 | 9.8 | 0.85 |
| **Nid7** | $C_{11}H_{14}O_3$ | 193.08716 | 16.17 | 163.041; 178.065; 135.046; 89.023; 44.998 | 7.61 | 0.67 |
| **Nid8** | | 193.08703 | 16.75 | 163.040; 178.064; 135.046; 55.018 | >30 | 0.05 |
| **Nid9** | $C_{18}H_{30}O_8$ | 373.18606 | 10.41 | 59.014; 161.046; 191.056; 331.178; 119.033 | 27.0 | 2.92 |
| **Nid10** | | 373.18538 | 13.13 | 59.014;331.177; 161.046; 101.025;113.024; 89.023 | >30 | 2.87 |
| **Nid11** | $C_{19}H_{26}O_{10}$ | 413.14386 | 11.55 | 269.105; 99.046; 59.014; 57.035; 125.025; | 27.7 | 3.24 |
| **Nid12** | | 413.14402 | 13.00 | 107.050; 99.045; 57.035; 101.025; 125.024 | 24.9 | 2.20 |
